# Genetic affinities of an eradicated European *Plasmodium falciparum* strain

**DOI:** 10.1099/mgen.0.000289

**Published:** 2019-08-27

**Authors:** Toni de-Dios, Lucy van Dorp, Pere Gelabert, Christian Carøe, Marcela Sandoval-Velasco, Rosa Fregel, Raül Escosa, Carles Aranda, Silvie Huijben, François Balloux, M. Thomas P. Gilbert, Carles Lalueza-Fox

**Affiliations:** ^1^ Institute of Evolutionary Biology (CSIC-UPF), 08003 Barcelona, Spain; ^2^ UCL Genetics Institute, University College London, Gower Street, London WC1E 6BT, UK; ^3^ Section for Evolutionary Genomics, Department of Biology, University of Copenhagen, 1353 Copenhagen, Denmark; ^4^ Department of Genetics, Stanford University, Stanford, CA, USA; ^5^ Department of Biochemistry, Microbiology, Cell Biology and Genetics, Universidad of La Laguna, 38206 La Laguna, Spain; ^6^ Consorci de Polítiques Ambientals de les Terres de l'Ebre (COPATE), 43580 Deltebre, Spain; ^7^ Servei de Control de Mosquits, Consell Comarcal del Baix Llobregat, 08980 Sant Feliu de Llobregat, Spain; ^8^ Center for Evolution and Medicine, School of Life Sciences, Arizona State University, Tempe, AZ 85281, USA; ^9^ Norwegian University of Science and Technology (NTNU) University Museum, N-7491 Trondheim, Norway

**Keywords:** malaria, *Plasmodium falciparum*, ancient genomics, drug resistance

## Abstract

Malaria was present in most of Europe until the second half of the 20th century, when it was eradicated through a combination of increased surveillance and mosquito control strategies, together with cross-border and political collaboration. Despite the severe burden of malaria on human populations, it remains contentious how the disease arrived and spread in Europe. Here, we report a partial *Plasmodium falciparum* nuclear genome derived from a set of antique medical slides stained with the blood of malaria-infected patients from Spain’s Ebro Delta, dating to the 1940s. Our analyses of the genome of this now eradicated European *P. falciparum* strain confirms stronger phylogeographical affinity to present-day strains in circulation in central south Asia, rather than to those in Africa. This points to a longitudinal, rather than a latitudinal, spread of malaria into Europe. In addition, this genome displays two derived alleles in the *pfmrp1* gene that have been associated with drug resistance. Whilst this could represent standing variation in the ancestral *P. falciparum* population, these mutations may also have arisen due to the selective pressure of quinine treatment, which was an anti-malarial drug already in use by the time the sample we sequenced was mounted on a slide.

## Data Summary

*Plasmodium falciparum* reads from Ebro-1944 have been deposited in the European Nucleotide Archive under accession number ERP114811. All modern *P. falciparum* samples used for the population genomics analyses were reported by the MalariaGEN *Plasmodium falciparum* Community Project in 2016 [1] and are provided in Table S1 (available in the online version of this article). All software used in the bioinformatic analyses are publicly available. Positions screened for anti-malarial drug resistance are available in Table S2.

**Impact Statement**

Malaria is a serious infectious disease affecting over 200 million people annually. The disease is caused by species of parasitic protozoans from the genus *Plasmodium*, which are transmitted by several species of mosquitoes from the genus *Anopheles*. Today, *Plasmodium* is restricted to tropical and subtropical latitudes. However, malaria was historically present in most of Europe, spanning from southern Britain and the Mediterranean to as far north as Finland. Spain represented one of its last footholds, where it persisted until the 1960s. Here, we report a substantial fraction of the genome of a 20th century European *Plasmodium falciparum* strain isolated from slides stained with the blood of malaria-infected patients in the 1940s. We analyse this genome in the context of worldwide modern strains to trace the historical dispersal of *P. falciparum* in Europe. We find evidence supporting a longitudinal spread from Asia into Europe, over a latitudinal spread from Africa, as well as variants in anti-malarial resistance genes predating the use of most common anti-malarial drugs. Our work highlights the potential of collections of antique medical slides to open new possibilities in the study of ancient microbial genomics, including malaria.

## Introduction

Classical Greek accounts in the fourth and fifth centuries BCE describe people with intermittent fevers and infectious symptoms characteristic of malaria [2]. The Roman author Celsius was able to accurately differentiate the clinical symptoms of *Plasmodium vivax* versus *Plasmodium malariae* infections [3]. In contrast, it is unclear whether *Plasmodium falciparum*, the deadliest form of the pathogen, was already present in classical times. While some authors argue that *P. falciparum* only spread to southern Europe with the dawn of the Roman Empire, historical accounts suggest it may have affected western and central Italy as early as 400–100 BCE [4], before reaching the Po Delta region of northern Italy around 1000 years later [3]. The spread of this pathogen through Italy in historical times is supported by the discovery of a large infant cemetery at Lugnano (Teverina, Umbria, Italy), dating to approximately 450 CE, and the report of *P. falciparum* ribosomal DNA sequences obtained from one of these skeletons by means of traditional PCR [5]. The recent retrieval of larger amounts of *P. falciparum* genetic data, by means of capture baits and second-generation sequencing technologies, from teeth sampled in Velia and Vagnari cemeteries (Italy), directly places this parasite in southern Italy by the beginning of the Roman Imperial period [6].

Genetic analyses of DNA recovered from a unique set of antique microscopy slides (1942–1944), stained with the blood of malaria-infected patients in the Ebro Delta of Spain for immediate diagnostic purposes, allowed us to report the complete mitochondrial DNA (mtDNA) genomes of historical *P. falciparum* and *P. vivax* [7]. In this study, we analyse data generated through merging the sequence information derived from four different slides, allowing us to reconstruct the partial nuclear genome of this eradicated European *P. falciparum* strain.

## Methods

### Sample collection

Four microscope slides, dated between 1942 and 1944, were selected for this study. All were obtained from Dr Ildefonso Canicio’s family collection. Dr Canicio was in charge of the anti-malarial hospital at Sant Jaume d’Enveja (Ebro Delta, Spain), inaugurated in 1925, until his death in 1961. Patients were predominantly local people who worked in the Ebro rice fields and had no history of international travel. The samples consist of four labelled microscopy slides stained with Giemsa – probably not previously fixed – in which parasites were still visible under the microscope. DNA extraction was performed in dedicated ancient DNA (aDNA) laboratories at the Institute of Evolutionary Biology in Barcelona (Spain) and the Centre for GeoGenetics in Copenhagen (Denmark) in 2015 and 2017, respectively, as described in the Supplementary Material.

### Ancient sample mapping and assembly

We first analysed the sequenced reads obtained from our eradicated European *P. falciparum* samples using *FastQC* (v0.11.7) [8] in order to determine their quality before and after trimming of the adapter sequences. We removed sequencing adapters and reads shorter than 30 bp using *cutadapt* (v1.3) [9]. We then mapped our reads against the *P. falciparum* 3D7 and *P. vivax* Sal1 reference genomes using BWA (v0.7.17) [10] aln, specifying no seeding, a gap open penalty of 2, an edit distance of 0.01 and no trimming; parameters shown to optimize mapping of ancient microbial samples [11]. We removed duplicated reads using *Picard* (v2.18.6) ‘MarkDuplicates’ and retained all mapped reads with a map quality of at least 30 in *SAMtools* (v1.6) [12]. As the blood samples had known co-infection with *P. vivax*, we extracted the sequencing reads mapping more confidently to *P. falciparum* by selecting those reads that had a lesser edit distance between *P. falciparum* 3D7 than with *P. vivax* Sal1 (Fig. S1). The obtained G+C content of the reads matched the expected and extremely low G+C content characteristic of the *P. falciparum* genome (Fig. S2).

Post-mortem aDNA damage patterns were determined using *MapDamage* (v2.0.8) [13] (Fig. S3). Most of the *P. falciparum* reads (0.6294× mean coverage, representing 88 % of the total genome) come from a single slide (labelled CA) sequenced in 2015. The remaining reads come from three other slides with mean genome-wide coverage of 0.0213×, 0.0285× and 0.0314×. The reduced coverage obtained from these slides relates to the overall endogenous sequence content and quality, which may also vary depending on the stage of the parasite’s life cycle when the slides were prepared. To increase the overall coverage, we therefore merged data from all four slides, always calling the dominant allele. We call our resultant composite genome Ebro-1944.

We also generated a reference panel of modern *P. falciparum* samples from publicly available sequence data [1]. In all cases, we mapped reads using *BWA* (v0.7.17) [10] *mem*, before removing duplicated sequence reads and filtering by mapping quality using thresholds as described above. The same procedure was followed for mapping the genome of *Plasmodium praefalciparum*, which was used as an outgroup. *Qualimap* (v2.2) [14] was used to generate the mapping metrics for all samples.

### Variant calling and dataset creation

To compare Ebro-1944 to current strains in global circulation, we selected modern strains with a mean depth of coverage equal or above 50× and with at least 90 % of the reference genome covered. This filtering strategy resulted in a dataset comprising 206 global samples. We used *GATK* (v3.7) [15], algorithm *UnifiedGenotyper*, to call variants, specifying *EMIT_ALL_CONFIDENT_SITES* and a standard confidence threshold over 50. We subsequently used *VCFtools* (0.1.14) [16] to filter out positions with less than 40× coverage, heterozygous positions, multi-allelic positions, indels and recombinant sub-telomeric regions (see the Supplementary Material). These samples were later merged using *bcftools* (v1.3.1) [12] and all variants that were not present in at least three samples were removed leaving 681 486 single nucleotide positions (SNPs). We used these positions to call additional modern samples, which mapped with lower coverage (<50×), applying the same filters. The resultant calls were merged using *bcftools*. SNPs were called for *P. praefalciparum* using the same parameters.

For Ebro-1944, we used a different approach to overcome the post-mortem damage associated with aDNA samples. We generated pseudo-haploid calls at the positions identified in the population genetics dataset using *SAMtools* to call a random base drawn from all possible reads at each site [17]. We then merged all the samples (including Ebro-1944) using *bcftools*. The complete dataset was filtered for a minimal minor allele frequency of 0.01 and multi-allelic SNPs were removed using *VCFtools*. All SNPs that were not present in at least 75 % of the samples considered were also removed. This procedure resulted in a final dataset comprising 435 samples and 14 346 bi-allelic SNPs, with 50.85 % of these covered in Ebro-1944.

### Population genetics analyses

This dataset was compiled by filtering all modern samples for sites called in Ebro-1944 and retaining all samples that had at least 50 % of these sites covered. This resulted in a dataset of 306 samples and 6 755 SNPs. A principal component analysis (PCA) was performed on this dataset using SmartPCA within the *EIG* (v.6.0.1) suite of tools [18].

To formally test the relative affinity of Ebro-1944 to South Asian, Oceanian and African strains, we used the full 14 346 biallelic SNP dataset to generate *f*4 statistics of the form *f*4(*P. praefalciparum*, Ebro-1944; X, Y), where we use *P. praefalciparum* as an outgroup and iterate through all combinations of geographical samples (X and Y) included in our global dataset. This statistic is designed to quantify the covariance in allele frequency differences between *P. praefalciparum* and Ebro-1944, and X and Y. If *P. praefalciparum* and Ebro-1944 form a clade with respect to X and Y, then their allele frequency differences should be uncorrelated and *f*4 will have a value of 0. Deviations from 0, thus, provide the relative affinity of Ebro-1944 to X and Y; positive values indicating a closer relationship to Y relative to X and negative values indicating a closer relationship to X relative to Y. *f*4 statistics were generated in qpDstat of *AdmixTools* (v.5.0) [19] and statistical significance was assessed through Z-scores following block jack-knife resampling (Fig. S4).

We additionally sub-sampled the global population genetics dataset more stringently, retaining only the strains with no missingness across the entire alignment, which led to a dataset of 30 strains over 8195 sites (Table S1). We generated patterns of pairwise haplotype sharing across this dataset using Chromopainter v2 [20], specifying a uniform recombination rate of 9.6 kb cM^−1^ [21] (Fig. S5) and using default estimates of mutation and switch parameters.

We also considered the mitochondrial relationships by placing our Ebro-1944 mtDNA genome (16.1×) in a minimum spanning network together with 435 global strains (Fig. S6). The mtDNA genome was also screened for specific variants restricted to South Asian strains.

### Resistance variants screening

A set of variants previously reported in the literature as associated with resistance to different anti-malarial drugs (Table S2) was screened in Ebro-1944 [22–46]. The position of the derived allele was determined using *Jvarkit* (v.2018.04.05) [47].

## Results

Our sequencing of the four antique microscopy slide samples yielded 218 952 *P*. *falciparum* reads producing a nuclear genome of 0.67× mean depth and covering 40.99 % of the *P. falciparum* reference genome 3D7 (Fig. S1). In addition, 1398 reads mapped to the mtDNA genome, generating a mean coverage of 16.1× over 99.43 % of the reference genome. Sequence reads showed damage patterns typical of aDNA (C to T and G to A substitutions at the 5′ and 3′ ends) in ratios of 3.8 and 2.2 %, respectively (Fig. S3). The characteristic aDNA damage of miscoding lesions indicates that our *P. falciparum* reads are authentically old, deriving from our historic specimen rather than from modern contamination where we would expect no such damage profile.

**Fig. 1.**
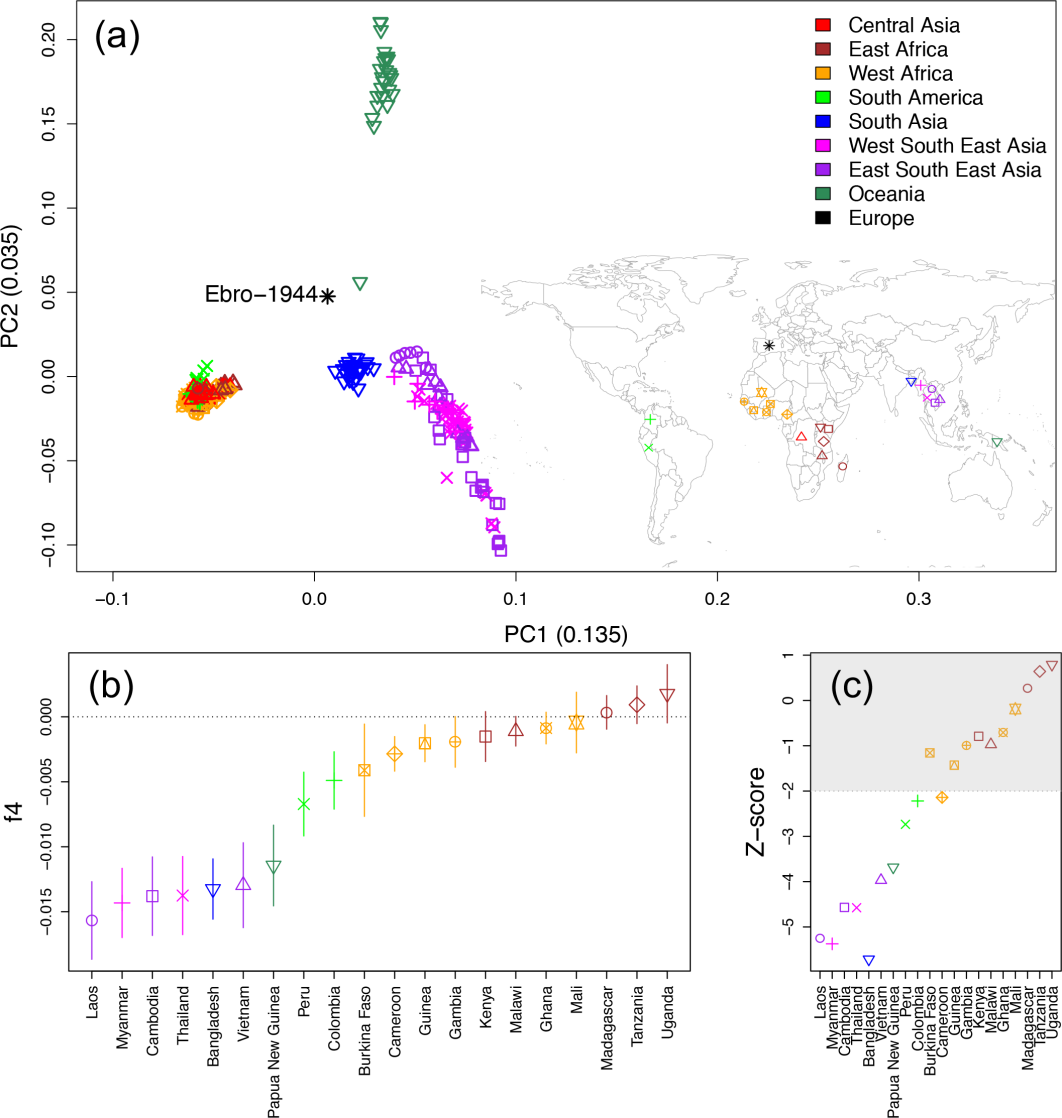
(a) PCA with worldwide sample locations (inset map). (b) *f*4-statistics under the test relationship *f*4(*P. praefalciparum*, Ebro-1944, X, Democratic Republic of Congo), where X iterates through the geographical sampling locations of our included modern *P. falciparum* strains. A more negative *f*4 value indicates a closer relationship of Ebro-1944 to X relative to strains sampled from the Democratic Republic of Congo. (c) Z-scores under the test relationship *f*4(*P. praefalciparum*, Ebro-1944, X, Colombia) assessed through block jack-knife resampling. All possible *f*4 topologies were tested (Fig. S4), with Ebro-1944 showing a consistent closer affinity to strains sampled from central south Asia.

PCA (Fig. 1a), *f*4 allele sharing statistics (Figs 1b, c, S4) and haplotype sharing analyses (Fig. S5) indicate that the closest genetic affinity of our European strain is to contemporary samples from central south Asia, including those currently in circulation in Laos, Myanmar and Vietnam. We also detect a shared genetic component with samples from Papua New Guinea, in particular PN0008-C (Fig. 1a). Whilst Ebro-1944 is more closely related to current strains from central south Asia than those from Africa, we found a higher proportion of African haplotypes in Ebro-1944 than in any central south Asian samples (two sample *t*-test *P*<2.2×10^−16^). Such a pattern would be consistent with a common origin of the European and central south Asian *P. falciparum* populations, followed by secondary introgression of African strains into the European population after the split between the European and central south Asian lineages (Fig. S5).

The close genetic affinity of the nuclear genome to strains in circulation in central south Asia is further confirmed at the mtDNA level (Fig. S6). Ebro-1944 carries two Indian-specific mtDNA substitutions at positions 276 and 2763 [48]. One additional mutation (position 725) is also present in our sample and is shared with nine contemporary Indian samples and two African samples included in our modern reference dataset (Table S1). We observe mutations at these three positions across all four of our sampled slides, suggesting the strains combined in Ebro-1944 are phylogenetically similar (Table S3). Unfortunately, these three mutations are not covered in a previously published partial strain retrieved from Roman cemetery sites [6]. Additionally, none of our reads overlapped with the ribosomal DNA fragment of European *P. falciparum* retrieved in a previous work [5].

We screened the Ebro-1944 nuclear genome for the presence of 117 variants that have been previously associated with anti-malarial drug resistance (Table S2). We achieved sequence coverage of at least one read at 62 positions. Of these, only two mutations (H191Y and I876V) displayed the resistance-associated allele (Table S2). Both alleles are located in the multidrug resistance protein 1 encoding gene (*pfmrp1)*, which encodes an ABC family transporter, and has been associated to alterations in quinine, chloroquine, artemisinin, piperaquine and primaquine sensitivity [49]. Although one of these mutations, H191Y, is a C to T substitution that is characteristic of post-mortem DNA deamination, and might, thus, be expected in ancient samples, we believe this to be authentic as the transition is present in two overlapping reads, and is not located at the end of the reads where damage tends to accumulate [50]. While the second variant, I876V, is only covered by a single read, it is an A to G substitution and is, thus, not a common DNA damage motif. To provide additional support for the presence of these derived alleles, we imputed these positions in our partial genome by using a reference panel of modern strains carrying the H191Y and I876V variants (see the Supplementary Material). Interestingly, while the latter variant is distributed worldwide, the former is currently restricted to Asia and Oceania, consistent with an Asian dispersal of *P. falciparum* into Europe.

## Discussion

We showed that an eradicated European *P. falciparum* strain from the 20th century is most closely related to extant strains from central south Asia, such as those currently in circulation in Laos, Myanmar and Vietnam. Although we detect some evidence for secondary introgression of African *P. falciparum* into the extinct European population, the significantly stronger affinity to Asian strains argues against a direct African origin [4] of European *P. falciparum*, and points instead to a migration event between Europe and Asia.

We cannot infer the directionality of the migration between Europe and Asia from the genetic evidence alone. A recent expansion of *P. falciparum* from Europe to Asia and Oceania, coinciding with European colonial expansion, might be conceivable given the well-characterized role of colonialism in the widespread dissemination of other major infectious diseases, such as *Mycobacterium tuberculosis* lineage 4, the globally distributed agent of tuberculosis [51].

However, a migration of *P. falciparum* from Europe to Asia does not sit well with historical evidence of the arrival of the parasite in Europe during antiquity [2–6]. Given that our extinct European strain shares significantly more alleles with extant Asian rather than African strains, the arrival of *P. falciparum* malaria parasites into Europe likely took place from the Asian sub-continent rather than spreading from Africa via the Mediterranean during the Roman Empire.

Plausible historical migrations responsible for a spread into Europe from the East include the extensive commercial exchanges and movement between people of various ethnicities in the Achaemenid Empire (550-330 BCE). Alternatively, *P. falciparum* might have reached Europe during the subsequent Hellenistic period, connecting India with the Mediterranean, following the conquest of the Achaemenid Empire by Alexander the Great.

The availability of a strain pre-dating most of the currently used anti-malarial drugs allowed us to look for the presence of resistance variants that may inform on the spread of such resistances in the future. The two resistance variants we observed in the *pfmrp1* gene could be explained by standing variation for drug-resistance mutations in *P. falciparum* or may have arisen following the use of quinine for over three centuries; chloroquines were not introduced in Spain until 1948 and initially only in the African colonies.

Our results provide novel insights into the evolution and past demography of one of the world’s deadliest pathogens, which could not have been reached by studying the genomes of extant strains alone. Additional genomic evidence from both medical collections and ancient remains will be needed to reconstruct more precise timings and routes for the spread of malaria into Europe, and could also help in determining the emergence and drivers of resistance to anti-malarial drugs.

## Data bibliography

1. The *Plasmodium falciparum* reference genome used was *P. falciparum* 3D7, assembly ASM276v2.

2. The *Plasmodium vivax* reference genome used was *P. vivax* Sal-1, assembly ASM241v2.

3. Details of the *Plasmodium falciparum* samples used in the population genetics analyses can be found under the study accession number PRJEB2136. Accession numbers for each sample are described in Table S1.

4. The sequence of *Plasmodium praefalciparum* can be found under accession number SAMEA2464702.

## Supplementary Data

Supplementary File 1

Supplementary File 2

## References

[1] MalariaGEN *Plasmodium falciparum* Community Project (2016). Genomic epidemiology of artemisinin resistant malaria. https://elifesciences.org/articles/08714.

[2] Jones WHS (1907). Malaria, a Neglected Factor in the History of Greece and Rome.

[3] Sallares R, Bouwman A, Anderung C (2004). The spread of malaria to southern Europe in antiquity: new approaches to old problems. Med Hist.

[4] De Zulueta J (1973). Malaria and Mediterranean history. Parassitologia.

[5] Sallares R, Gomzi S (2001). Biomolecular archaeology of malaria. Ancient Biomolecules.

[6] Marciniak S, Prowse TL, Herring DA, Klunk J, Kuch M (2016). *Plasmodium falciparum* malaria in 1st–2nd century CE southern Italy. Current Biology.

[7] Gelabert P, Sandoval-Velasco M, Olalde I, Fregel R, Rieux A (2016). Mitochondrial DNA from the eradicated European *Plasmodium vivax* and *P. falciparum* from 70-year-old slides from the Ebro Delta in Spain. Proc Natl Acad Sci USA.

[8] Andrews S (2010). http://www.Bioinformatics.Babraham.Ac.Uk/Projects/Fastqc/.

[9] Martin M (2011). Cutadapt removes adapter sequences from high-throughput sequencing reads.. EMBnet.Journal.

[10] Li H, Durbin R (2009). Fast and accurate short read alignment with Burrows-Wheeler transform. Bioinformatics.

[11] Key FM, Posth C, Krause J, Herbig A, Bos KI (2017). Mining metagenomic data sets for ancient DNA: recommended protocols for authentication. Trends Genet.

[12] Li H, Handsaker B, Wysoker A, Fennell T, Ruan J (2009). The Sequence Alignment/Map format and SAMtools. Bioinformatics.

[13] Jónsson H, Ginolhac A, Schubert M, Johnson PLF, Orlando L (2013). mapDamage2.0: fast approximate Bayesian estimates of ancient DNA damage parameters. Bioinformatics.

[14] García-Alcalde F, Okonechnikov K, Carbonell J, Cruz LM, Götz S (2012). Qualimap: evaluating next-generation sequencing alignment data. Bioinformatics.

[15] Van der Auwera GA, Carneiro MO, Hartl C, Poplin R, del Angel G (2013). From FastQ data to high-confidence variant calls: the Genome Analysis Toolkit best practices pipeline. Curr Protoc Bioinformatics.

[16] Danecek P, Auton A, Abecasis G, Albers CA, Banks E (2011). The variant call format and VCFtools. Bioinformatics.

[17] Green RE, Krause J, Briggs AW, Maricic T, Stenzel U (2010). A draft sequence of the Neandertal genome. Science.

[18] Price AL, Patterson NJ, Plenge RM, Weinblatt ME, Shadick NA (2006). Principal components analysis corrects for stratification in genome-wide association studies. Nat Genet.

[19] Patterson N, Moorjani P, Luo Y, Mallick S, Rohland N (2012). Ancient admixture in human history. Genetics.

[20] Lawson DJ, Hellenthal G, Myers S, Falush D (2012). Inference of population structure using dense haplotype data. PLoS Genet.

[21] Jiang H, Li N, Gopalan V, Zilversmit MM, Varma S (2011). High recombination rates and hotspots in a *Plasmodium falciparum* genetic cross. Genome Biol.

[22] Foote SJ, Kyle DE, Martin RK, Oduola AM, Forsyth K (1990). Several alleles of the multidrug-resistance gene are closely linked to chloroquine resistance in *Plasmodium falciparum*. Nature.

[23] Price RN, Cassar C, Brockman A, Duraisingh M, van Vugt M (1999). The *pfmdr1* gene is associated with a multidrug-resistant phenotype in *Plasmodium falciparum* from the Western border of Thailand. Antimicrob Agents Chemother.

[24] Dahlström S, Ferreira PE, Veiga MI, Sedighi N, Wiklund L (2009). *Plasmodium falciparum* multidrug resistance protein 1 and artemisinin-based combination therapy in Africa. J Infect Dis.

[25] Rottmann M, McNamara C, Yeung BKS, Lee MCS, Zou B (2010). Spiroindolones, a potent compound class for the treatment of malaria. Science.

[26] Setthaudom C, Tan-ariya P, Sitthichot N, Khositnithikul R, Suwandittakul N (2011). Role of *Plasmodium falciparum* chloroquine resistance transporter and multidrug resistance 1 genes on in vitro chloroquine resistance in isolates of *Plasmodium falciparum* from Thailand. Am J Trop Med Hyg.

[27] Veiga MI, Ferreira PE, Jörnhagen L, Malmberg M, Kone A (2011). Novel polymorphisms in *Plasmodium falciparum* ABC transporter genes are associated with major ACT antimalarial drug resistance. PLoS One.

[28] Takala-Harrison S, Clark TG, Jacob CG, Cummings MP, Miotto O (2013). Genetic loci associated with delayed clearance of *Plasmodium falciparum* following artemisinin treatment in Southeast Asia. Proc Natl Acad Sci USA.

[29] Gupta B, Xu S, Wang Z, Sun L, Miao J (2014). *Plasmodium falciparum* multidrug resistance protein 1 (pfmrp1) gene and its association with in vitro drug susceptibility of parasite isolates from north-east Myanmar. J Antimicrob Chemother.

[30] Vaidya AB, Morrisey JM, Zhang Z, Das S, Daly TM (2014). Pyrazoleamide compounds are potent antimalarials that target Na+ homeostasis in intraerythrocytic *Plasmodium falciparum*. Nat Commun.

[31] Miotto O, Amato R, Ashley EA, MacInnis B, Almagro-Garcia J (2015). Genetic architecture of artemisinin-resistant *Plasmodium falciparum*. Nat Genet.

[32] Pelleau S, Moss EL, Dhingra SK, Volney B, Casteras J (2015). Adaptive evolution of malaria parasites in French Guiana: reversal of chloroquine resistance by acquisition of a mutation in *pfcrt*. Proc Natl Acad Sci USA.

[33] Callaghan PS, Siriwardana A, Hassett MR, Roepe PD (2016). *Plasmodium falciparum* chloroquine resistance transporter (PfCRT) isoforms PH1 and PH2 perturb vacuolar physiology. Malar J.

[34] Reed MB, Saliba KJ, Caruana SR, Kirk K, Cowman AF (2000). Pgh1 modulates sensitivity and resistance to multiple antimalarials in *Plasmodium falciparum*. Nature.

[35] Mishra N, Bharti RS, Mallick P, Singh OP, Srivastava B (2016). Emerging polymorphisms in falciparum Kelch 13 gene in Northeastern region of India. Malar J.

[36] Wang Z, Cabrera M, Yang J, Yuan L, Gupta B (2016). Genome-wide association analysis identifies genetic loci associated with resistance to multiple antimalarials in *Plasmodium falciparum* from China-Myanmar border. Sci Rep.

[37] Ye R, Hu D, Zhang Y, Huang Y, Sun X (2016). Distinctive origin of artemisinin-resistant *Plasmodium falciparum* on the China-Myanmar border. Sci Rep.

[38] Kobasa T, Talundzic E, Sug-aram R, Boondat P, Goldman IF (2018). Emergence and spread of kelch13 mutations associated with artemisinin resistance in *Plasmodium falciparum* parasites in 12 Thai provinces from 2007 to 2016. Antimicrob Agents Chemother.

[39] Ross LS, Dhingra SK, Mok S, Yeo T, Wicht KJ (2018). Emerging Southeast Asian PfCRT mutations confer *Plasmodium falciparum* resistance to the first-line antimalarial piperaquine. Nat Commun.

[40] Mu J, Ferdig MT, Feng X, Joy DA, Duan J (2003). Multiple transporters associated with malaria parasite responses to chloroquine and quinine. Mol Microbiol.

[41] Pickard AL, Wongsrichanalai C, Purfield A, Kamwendo D, Emery K (2003). Resistance to antimalarials in Southeast Asia and genetic polymorphisms in PfMDR1. Antimicrob Agents Chemother.

[42] Durrand V, Berry A, Sem R, Glaziou P, Beaudou J (2004). Variations in the sequence and expression of the *Plasmodium falciparum* chloroquine resistance transporter (Pfcrt) and their relationship to chloroquine resistance in vitro. Mol Biochem Parasitol.

[43] Happi CT, Gbotosho GO, Folarin OA, Akinboye DO, Yusuf BO (2005). Polymorphisms in *Plasmodium falciparum* dhfr and dhps genes and age related in vivo sulfadoxine-pyrimethamine resistance in malaria-infected patients from Nigeria. Acta Trop.

[44] Sidhu ABS, Valderramos SG, Fidock DA (2005). Pfmdr1 mutations contribute to quinine resistance and enhance mefloquine and artemisinin sensitivity in *Plasmodium falciparum*. Mol Microbiol.

[45] Echeverry DF, Holmgren G, Murillo C, Higuita JC, Björkman A (2007). Polymorphisms in the *pfcrt* and *pfmdr1* genes of *Plasmodium falciparum* and in vitro susceptibility to amodiaquine and desethylamodiaquine. Am J Trop Med Hyg.

[46] Dahlström S, Veiga MI, Mårtensson A, Björkman A, Gil JP (2009). Polymorphism in PfMRP1 (*Plasmodium falciparum* multidrug resistance protein 1) amino acid 1466 associated with resistance to sulfadoxine-pyrimethamine treatment. Antimicrob Agents Chemother.

[47] Pierre L (2015). JVarkit: java-based utilities for bioinformatics.

[48] Tyagi S, Pande V, Das A (2014). New insights into the evolutionary history of *Plasmodium falciparum* from mitochondrial genome sequence analyses of Indian isolates. Mol Ecol.

[49] Raj DK, Mu J, Jiang H, Kabat J, Singh S (2009). Disruption of a *Plasmodium falciparum* multidrug resistance-associated protein (PfMRP) alters its fitness and transport of antimalarial drugs and glutathione. J Biol Chem.

[50] Briggs AW, Stenzel U, Johnson PLF, Green RE, Kelso J (2007). Patterns of damage in genomic DNA sequences from a Neandertal. Proc Natl Acad Sci USA.

[51] Brynildsrud OB, Pepperell CS, Suffys P, Grandjean L, Monteserin J (2018). Global expansion of *Mycobacterium tuberculosis* lineage 4 shaped by colonial migration and local adaptation. Sci Adv.

