# Supplementary material for: Genetic affinities of an eradicated European Plasmodium falciparum strain
Source: Microb Genom. 2019 Aug 27;5(9):e000289. doi: 10.1099/mgen.0.000289 (PMC6807384; doi:10.1099/mgen.0.000289)
Supplement: Supplementary File 1 [file mgen-5-289-s001.pdf]

Supplementary Materials

DNA extraction

The slides were immersed in 10 –15 mL of lysis buffer (consisting of 0,5% SDS, 0,25 mg/mL proteinase K, 10 mM Tris and 0.5 M EDTA pH 8.0) and left in 50 mL Falcon tubes at 37ºC overnight. In order to protect the written labels (if present), one of the ends remained unsubmerged. Afterwards, the supernatant was concentrated using a silica column-based method, as described by a protocol used to recover short and highly degraded DNA fragments from very ancient samples (1).

Library preparation and amplification

Double stranded libraries were created using NEBNext DNA Sample Prep Master Mix Set 2 (E6070; New England Biolabs) following the manufacturer's instructions with Illumina adapters as described in Dabney *et al.* 2013 (1). We determined the optimal number of required cycles necessary to amplify the samples and thus obtain a suitable amount of DNA (100-500 ng) using quantitative (q)PCR.

Capture depletion

As the expected quantity of *Plasmodium* DNA present in the slides is minimal in comparison to the more abundant human DNA from the host's cells, we used the following procedures: we first tried to reduce the human DNA content through whole genome capture with human baits and shotgun-sequenced the waste product. Additionally, we carried out a capture enrichment approach using whole genome baits synthesized for *P. falciparum* genomic (g)DNA, as described in March *et al.* 2013 (2). Genomic DNA obtained from the *P. falciparum* African strain 3D7 in *in vitro* culture (MRA-102G, MR4; ATCC) was fragmented and built into different libraries with a T7 adapter incorporated. These *Plasmodium* T7 libraries were subsequently used to generate biotinylated RNA baits by *in vitro* transcription. The capture-depletion assay

using whole-genome human baits was done following Mybait Human Whole genome to manual version 3.01 (from www.microarray.com/pdf/Mybaits-manual-v3.pdf). After hybridization of the ancient (a)DNA libraries with the human baits for 24 hours, we let it bind to streptavidin magnetic beads for 30 minutes at 65 ºC. Finally, we collected the supernatant (fraction that did not bind to the beads) and cleaned it using QiaQuick PCR Purification Kit (Qiagen) following the manufacturer's instructions. The samples were eluted in 30 µl of Elution Buffer (EB, Qiagen) after 10-minutes of incubation at 37 ºC.

Amplification of capture-depleted products

After we had estimated the optimal number of cycles with qPCR, capture-depletion products were amplified for five cycles and *P. falciparum* DNA reads captured for 22 cycles using 2x KAPA HotStart ReadyMix and re-amplification primers IS5 and IS6 (3). The samples were then quantified on an Agilent 2100 Bioanalyzer (Agilent technologies) and pooled in equimolar amounts. The pool was sequenced in one lane of an Illumina HiSEQ 4500 run in 80 SR mode. A library blank and an extraction blank control were included and showed no evidence of contamination with exogenous *P. falciparum* DNA. This extraction and amplification process resulted in us using all available material and slides.

Reference dataset

In order to represent the global *P. falciparum* diversity, we selected 434 worldwide *P. falciparum* samples from Amato et al 2016 (4). The final population genetics dataset consisted of 58 samples from Central Africa, 62 from East Africa, 62 from West Africa, 27 from South America, 48 from South Asia, 70 from West South East Asia, 64 from East South East Asia and 43 from Oceania. The full list of countries, samples and identifiers represented in each group can be found in Amato et al 2016 (4). We also selected a *P. praefalciparum* genome (accession code ERS437570) as an outgroup species (5). We detail the dataset in Supplementary Table S1.

Highly recombinant genes

In order to reduce the noise produced by highly variable recombinant regions, we removed a set of sub-telomeric genes at positions described in the literature (6–20).

Mitochondrial analysis

We extracted only the mtDNA alignment of the population genetics dataset, which comprised 5967 bp and 251 SNPs across 426 samples, including the *P. praefalciparum* outgroup. A pairwise similarity matrix was constructed based on the raw SNP differences and used to generate a minimum spanning network using the spantree() function from the R package Vegan(21).

P. falciparum 18s rDNA sequence

We compared the reads mapped against the 18s rDNA gene with 2 previously published sequences of the same gene (22). Using BLASTn, both published sequences showed 100% identity with the 18s rDNA of *P. falciparum* (23). Unfortunately, our Ebro-1944 sequence did not overlap with this specific genetic region, but showed a high degree of identity with *P. falciparum* sequences (>95%).

Imputation of H191Y and I876V genetic variants at the *pfmrp1* gene

Given the low coverage of the Ebro-1944 nuclear genome, we conducted additional analyses to validate the presence and absence of variants involved in anti-malarial drug resistance. We performed imputation over a 100kb window of the genome containing the *pfmrp1* gene, and used GATK *UnifiedGenotyper* to call variants in this region (24). We then selected all samples with more than 80% of the positions called at a depth of 20x. We filtered out all the positions with indels and with a minor allele count below 2; and retained only the biallelic SNPs. This resulted in a dataset of 183 samples and 478 SNPs with no missing genotypes. This dataset was used as a reference for the imputation of Ebro-1944 using Beagle v.4.1 (25). The imputed results confirm the

presence of the derived allele at the positions chr_1:465296 (H191Y) and chr_1:467351 (I876V).

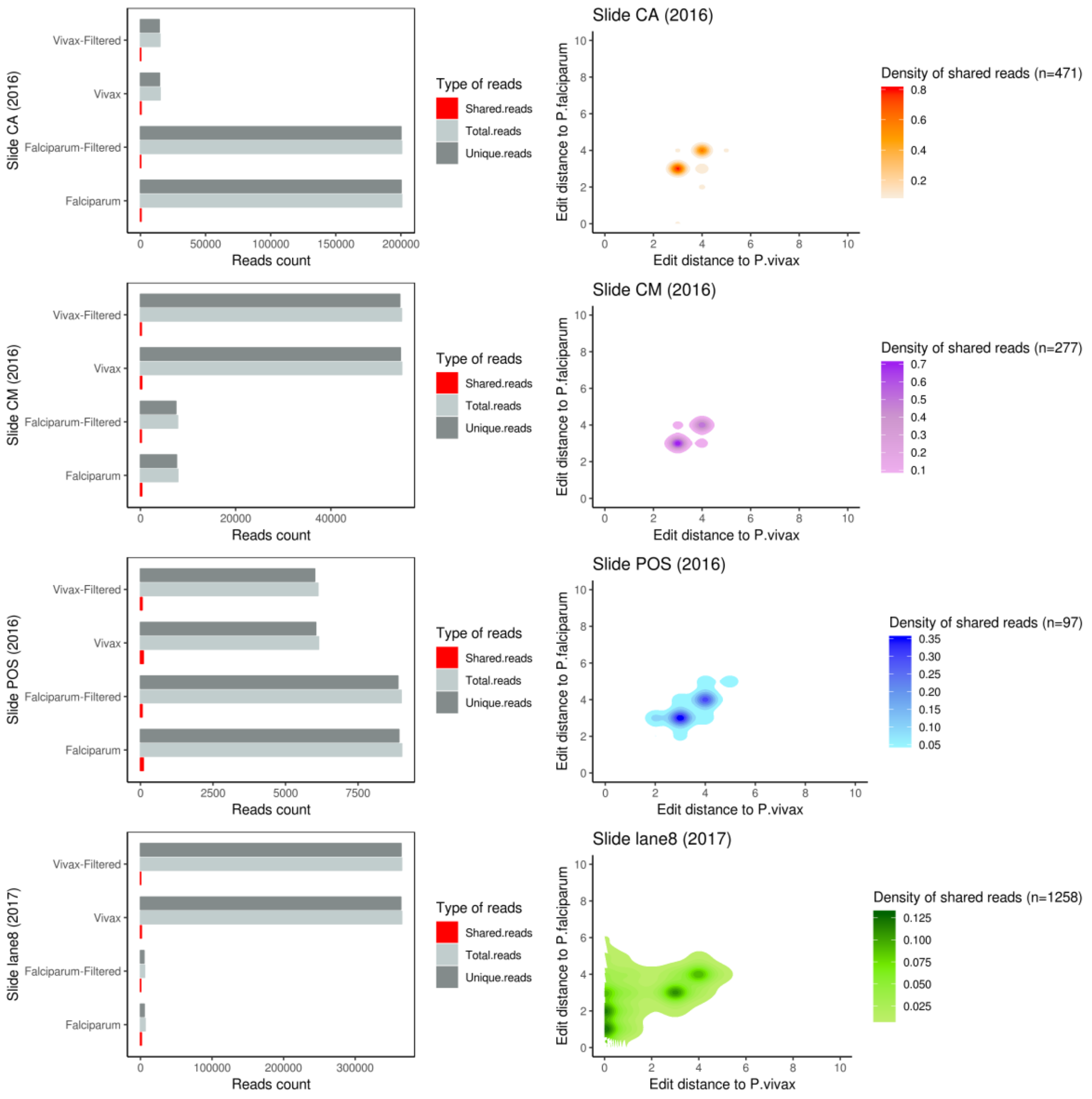

Figure S1: Comparison of read edit distance. Total amount (count) of reads mapped to *P. falciparum* and *P. vivax* in each slide (left), and comparison of the edit distance between shared reads mapped against the *P. falciparum* and *P. vivax* reference genomes (right). The majority of reads were obtained from Slide CA (top row).

A

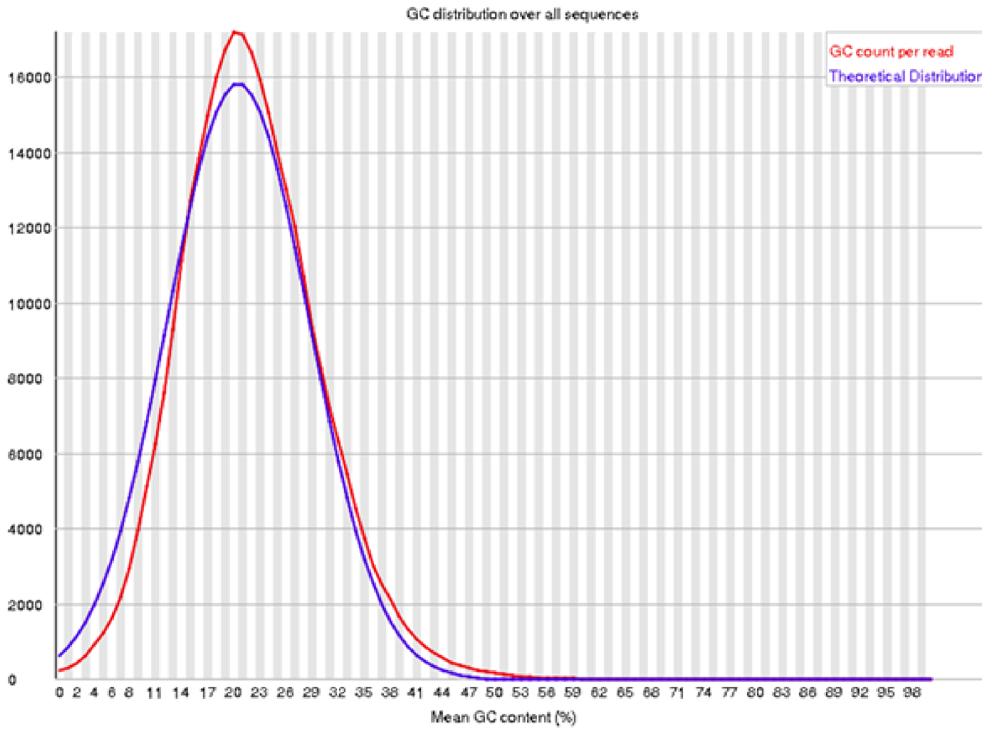

GC distribution over all sequences
GC count per read
Theoretical Distribution
16000
14000
12000
10000
8000
6000
4000
2000
0
0 2 4 6 8 11 14 17 20 23 26 29 32 35 38 41 44 47 50 53 56 59 62 65 68 71 74 77 80 83 86 89 92 95 98
Mean GC content (%)

B

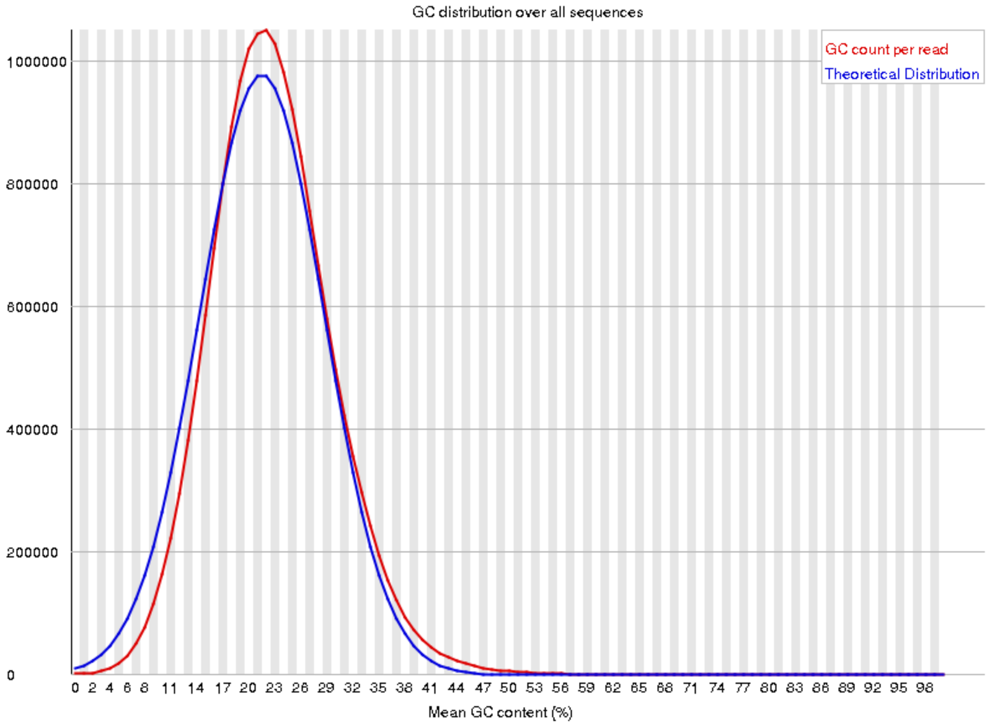

GC distribution over all sequences
GC count per read
Theoretical Distribution
1000000
800000
600000
400000
200000
0
0 2 4 6 8 11 14 17 20 23 26 29 32 35 38 41 44 47 50 53 56 59 62 65 68 71 74 77 80 83 86 89 92 95 98
Mean GC content (%)

C

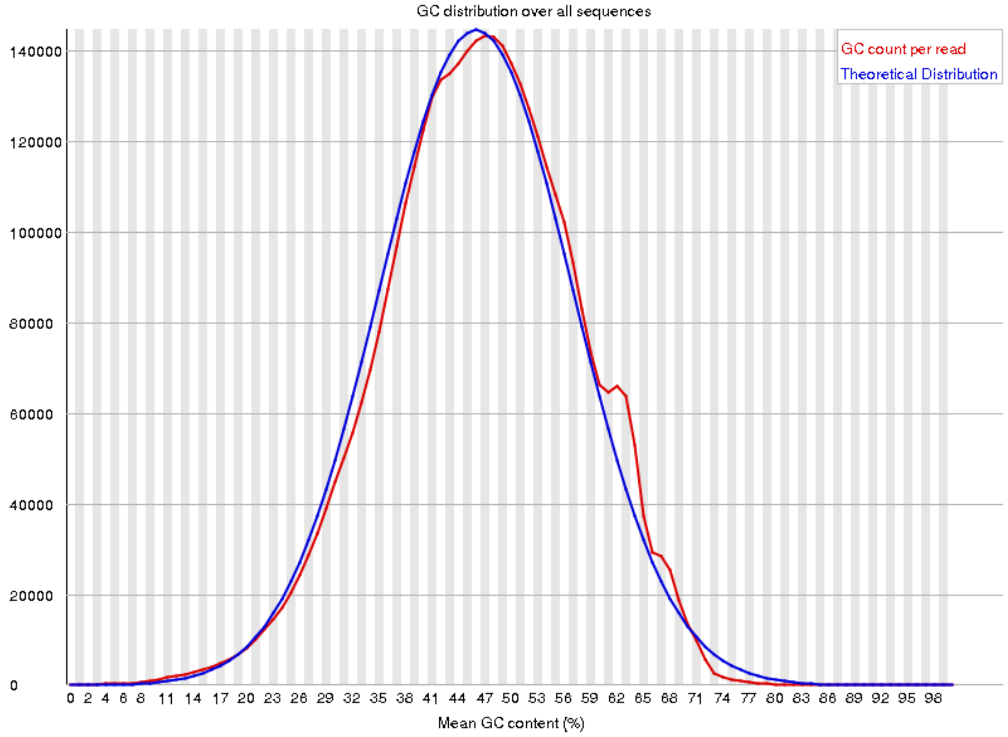

Figure S2: GC content. GC content of Ebro-1944 mapped against *3D7* (A). The y-axis provides the number of reads with an observed GC content (x-axis) in Ebro-1944 (red) and their theoretical distribution (blue). Figures S2 B and C provide the same profile for an example of modern *P. falciparum* (PR0124) and an example of modern *P. vivax* (PNG 030 sample), respectively (4,26).

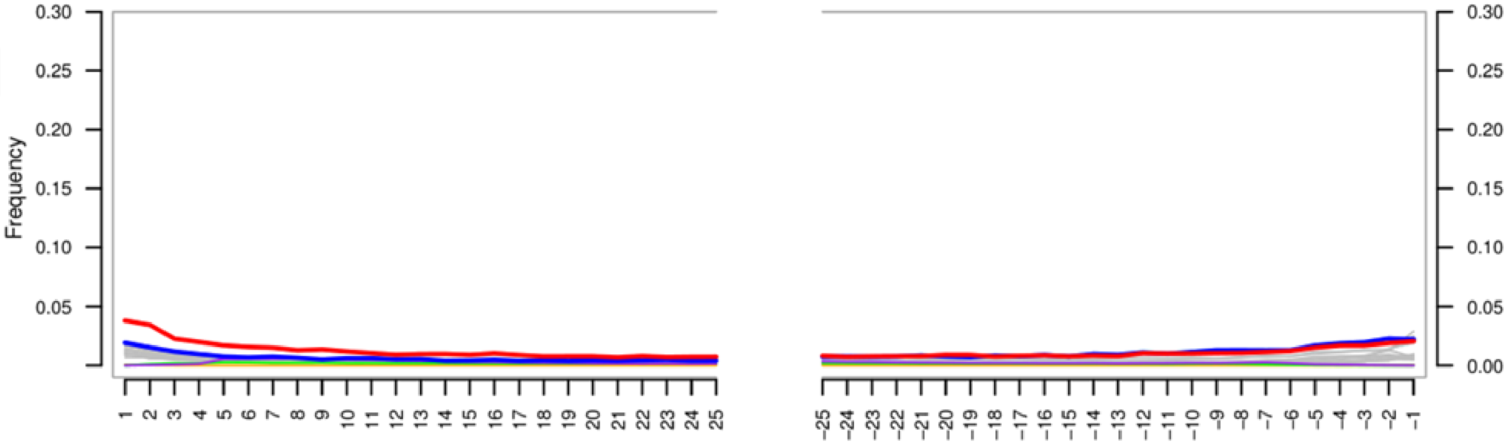

Figure S3: Post-mortem damage profiles of Ebro-1944 reads. The specific nucleotide positions (x-axis) at which a substitution is present at the 5’ end (left) and 3’ end (right) of the mapped reads is provided. In red, the C to T substitution frequency; in blue, the G to A substitution frequency; in grey, the frequency of all other substitutions. The elevation in C to T substitutions at the 5’ end and G to A substitutions at the 3’ end suggest DNA damage in Ebro-1944 is consistent with the degradation expected in post-mortem historical samples rather than modern contamination.

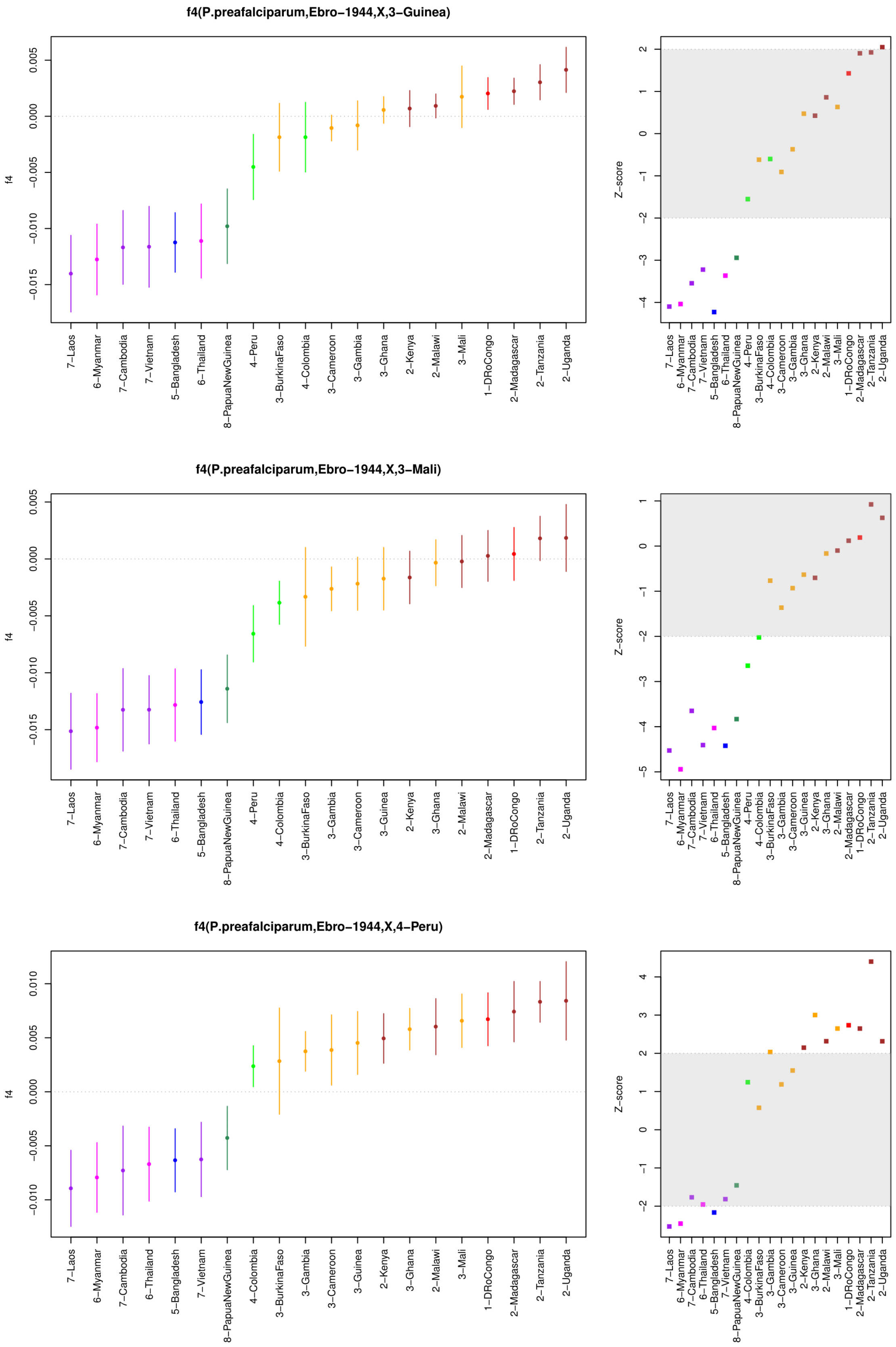

f4(P.preafalciparum,Ebro−1944,X,3−Guinea)
f4
Z−score
7−Laos
6−Myanmar
7−Cambodia
7−Vietnam
5−Bangladesh
6−Thailand
8−PapuaNewGuinea
4−Peru
3−BurkinaFaso
4−Colombia
3−Cameroon
3−Gambia
3−Ghana
2−Kenya
2−Malawi
3−Mali
1−DRoCongo
2−Madagascar
2−Tanzania
2−Uganda
f4(P.preafalciparum,Ebro−1944,X,3−Mali)
f4
Z−score
7−Laos
6−Myanmar
7−Cambodia
7−Vietnam
6−Thailand
5−Bangladesh
8−PapuaNewGuinea
4−Peru
4−Colombia
3−BurkinaFaso
3−Gambia
3−Cameroon
3−Guinea
2−Kenya
3−Ghana
2−Malawi
2−Madagascar
1−DRoCongo
2−Tanzania
2−Uganda
f4(P.preafalciparum,Ebro−1944,X,4−Peru)
f4
Z−score
7−Laos
6−Myanmar
7−Cambodia
6−Thailand
5−Bangladesh
7−Vietnam
8−PapuaNewGuinea
4−Colombia
3−BurkinaFaso
3−Gambia
3−Cameroon
3−Guinea
2−Kenya
3−Ghana
2−Malawi
3−Mali
1−DRoCongo
2−Madagascar
2−Tanzania
2−Uganda

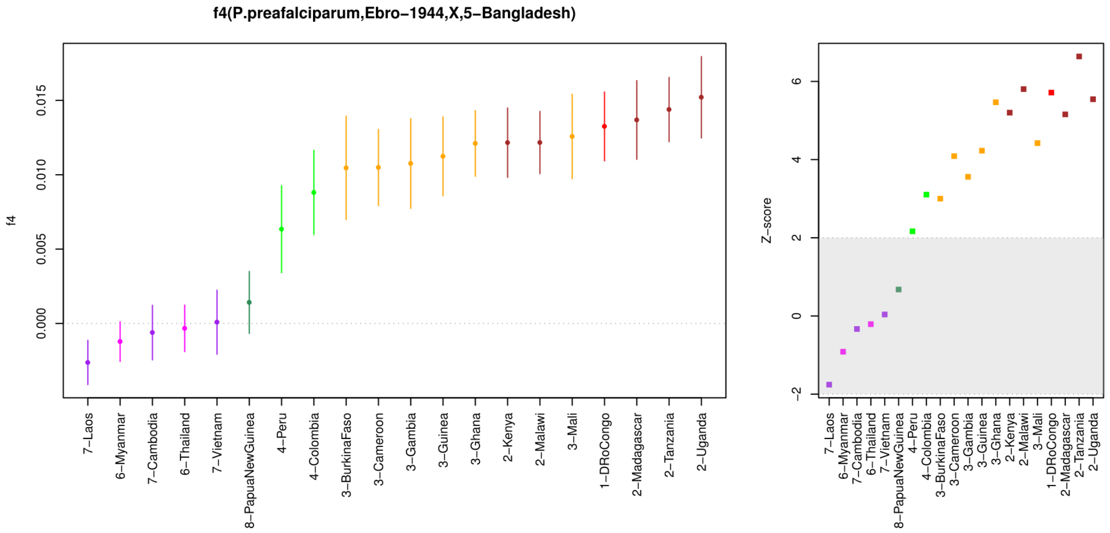
f4(P.preafalciparum,Ebro−1944,X,5−Bangladesh)
f4
Z−score
7−Laos
6−Myanmar
7−Cambodia
6−Thailand
7−Vietnam
8−PapuaNewGuinea
4−Peru
4−Colombia
3−BurkinaFaso
3−Cameroon
3−Gambia
3−Guinea
3−Ghana
2−Kenya
2−Malawi
3−Mali
1−DRoCongo
2−Madagascar
2−Tanzania
2−Uganda

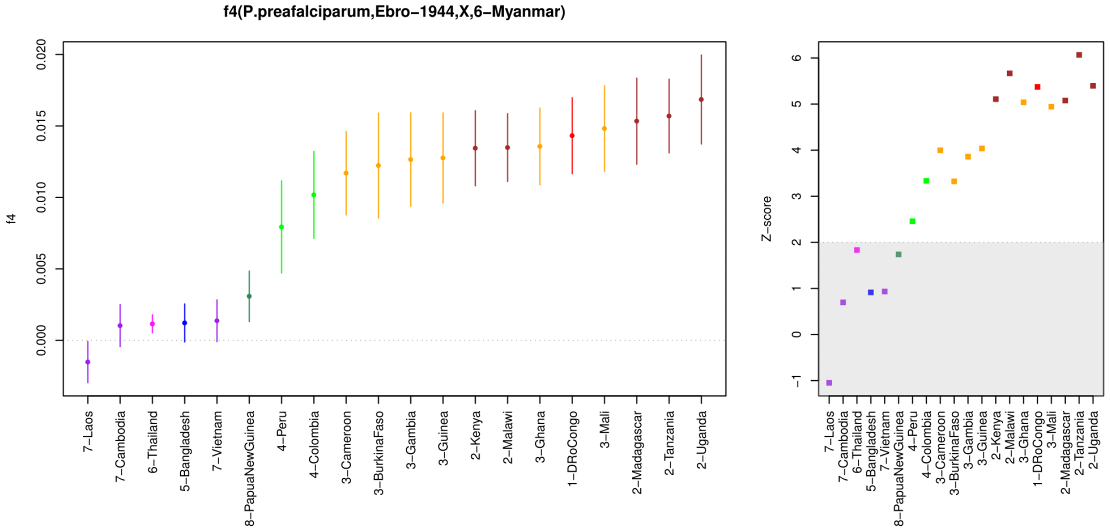
f4(P.preafalciparum,Ebro−1944,X,6−Myanmar)
f4
Z−score
7−Laos
7−Cambodia
6−Thailand
5−Bangladesh
7−Vietnam
8−PapuaNewGuinea
4−Peru
4−Colombia
3−Cameroon
3−BurkinaFaso
3−Gambia
3−Guinea
2−Kenya
2−Malawi
3−Ghana
1−DRoCongo
3−Mali
2−Madagascar
2−Tanzania
2−Uganda

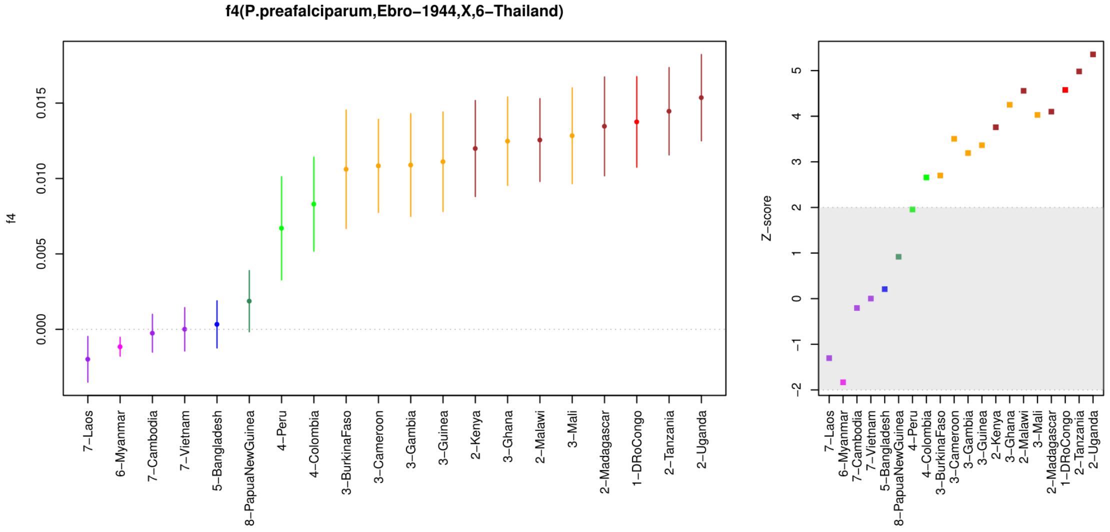
f4(P.preafalciparum,Ebro−1944,X,6−Thailand)
f4
Z−score
7−Laos
6−Myanmar
7−Cambodia
7−Vietnam
5−Bangladesh
8−PapuaNewGuinea
4−Peru
4−Colombia
3−BurkinaFaso
3−Cameroon
3−Gambia
3−Guinea
2−Kenya
3−Ghana
2−Malawi
3−Mali
2−Madagascar
1−DRoCongo
2−Tanzania
2−Uganda

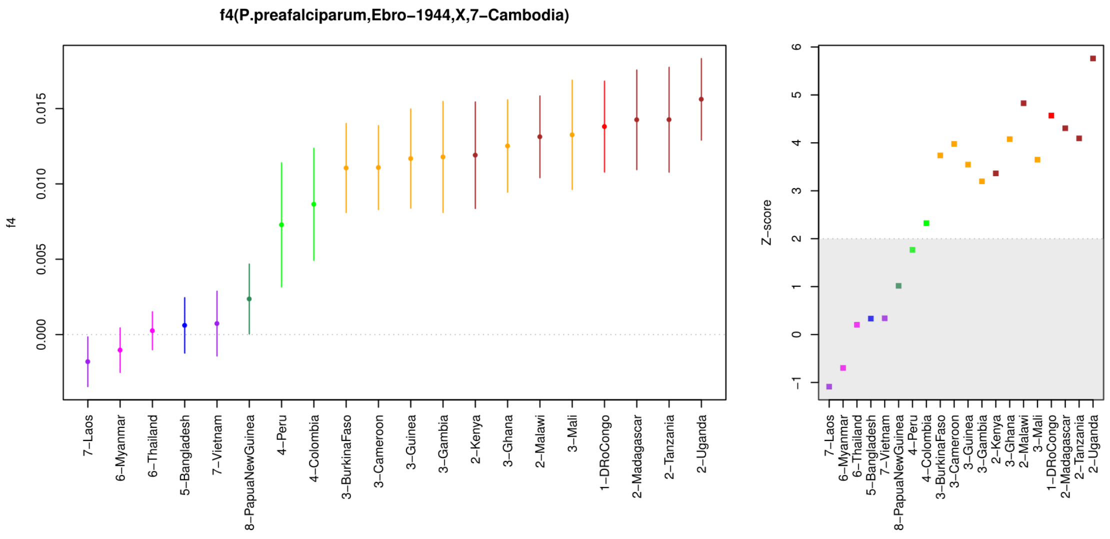
f4(P.preafalciparum,Ebro−1944,X,7−Cambodia)
f4
0.000
0.005
0.010
0.015
7−Laos
6−Myanmar
6−Thailand
5−Bangladesh
7−Vietnam
8−PapuaNewGuinea
4−Peru
4−Colombia
3−BurkinaFaso
3−Cameroon
3−Guinea
3−Gambia
2−Kenya
3−Ghana
2−Malawi
3−Mali
1−DRoCongo
2−Madagascar
2−Tanzania
2−Uganda
Z−score
−1
0
1
2
3
4
5
6

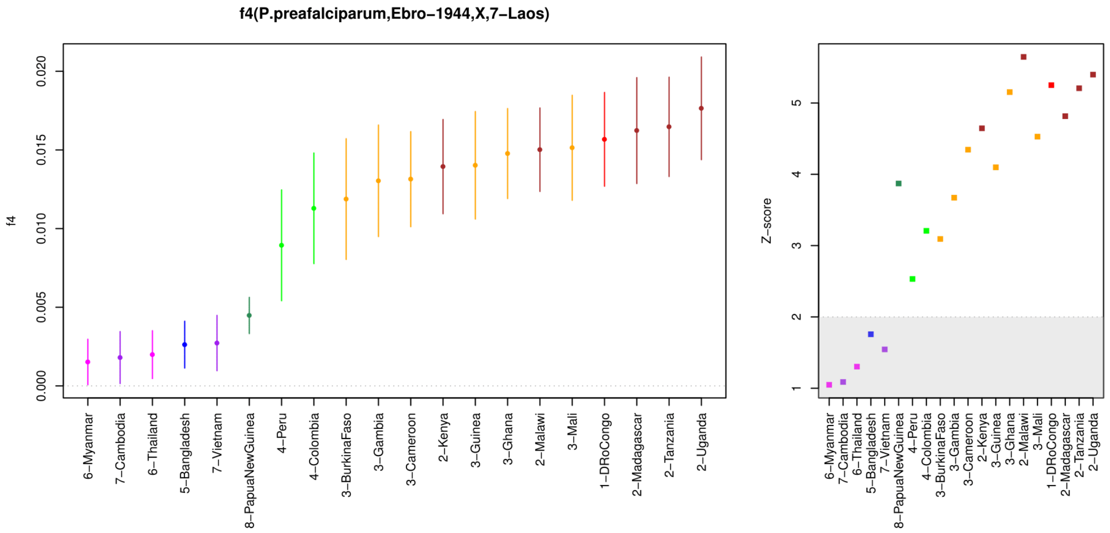
f4(P.preafalciparum,Ebro−1944,X,7−Laos)
f4
0.000
0.005
0.010
0.015
0.020
6−Myanmar
7−Cambodia
6−Thailand
5−Bangladesh
7−Vietnam
8−PapuaNewGuinea
4−Peru
4−Colombia
3−BurkinaFaso
3−Gambia
3−Cameroon
2−Kenya
3−Guinea
3−Ghana
2−Malawi
3−Mali
1−DRoCongo
2−Madagascar
2−Tanzania
2−Uganda
Z−score
1
2
3
4
5

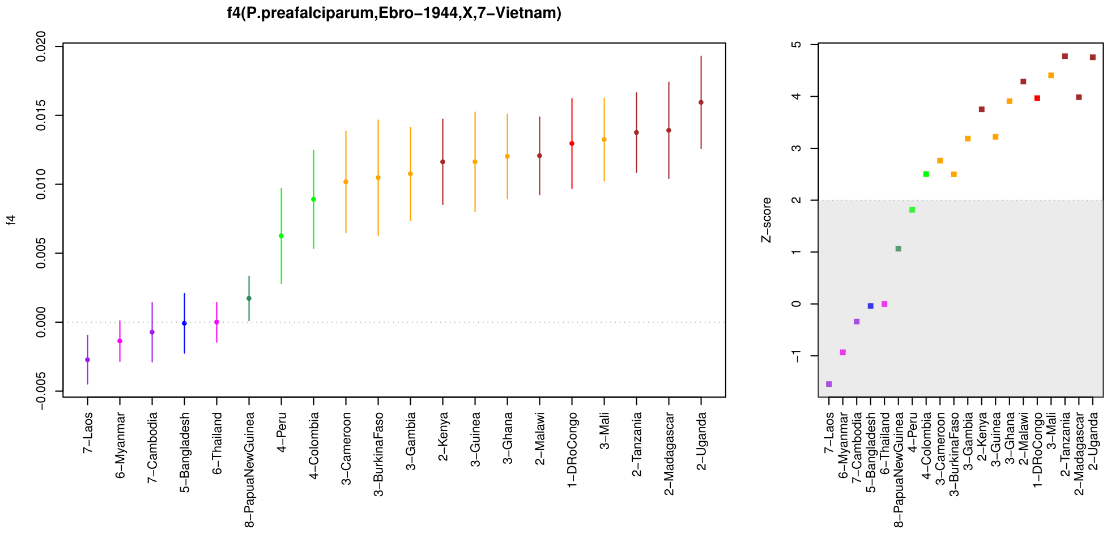
f4(P.preafalciparum,Ebro−1944,X,7−Vietnam)
f4
−0.005
0.000
0.005
0.010
0.015
0.020
7−Laos
6−Myanmar
7−Cambodia
5−Bangladesh
6−Thailand
8−PapuaNewGuinea
4−Peru
4−Colombia
3−Cameroon
3−BurkinaFaso
3−Gambia
2−Kenya
3−Guinea
3−Ghana
2−Malawi
1−DRoCongo
3−Mali
2−Tanzania
2−Madagascar
2−Uganda
Z−score
−1
0
1
2
3
4
5

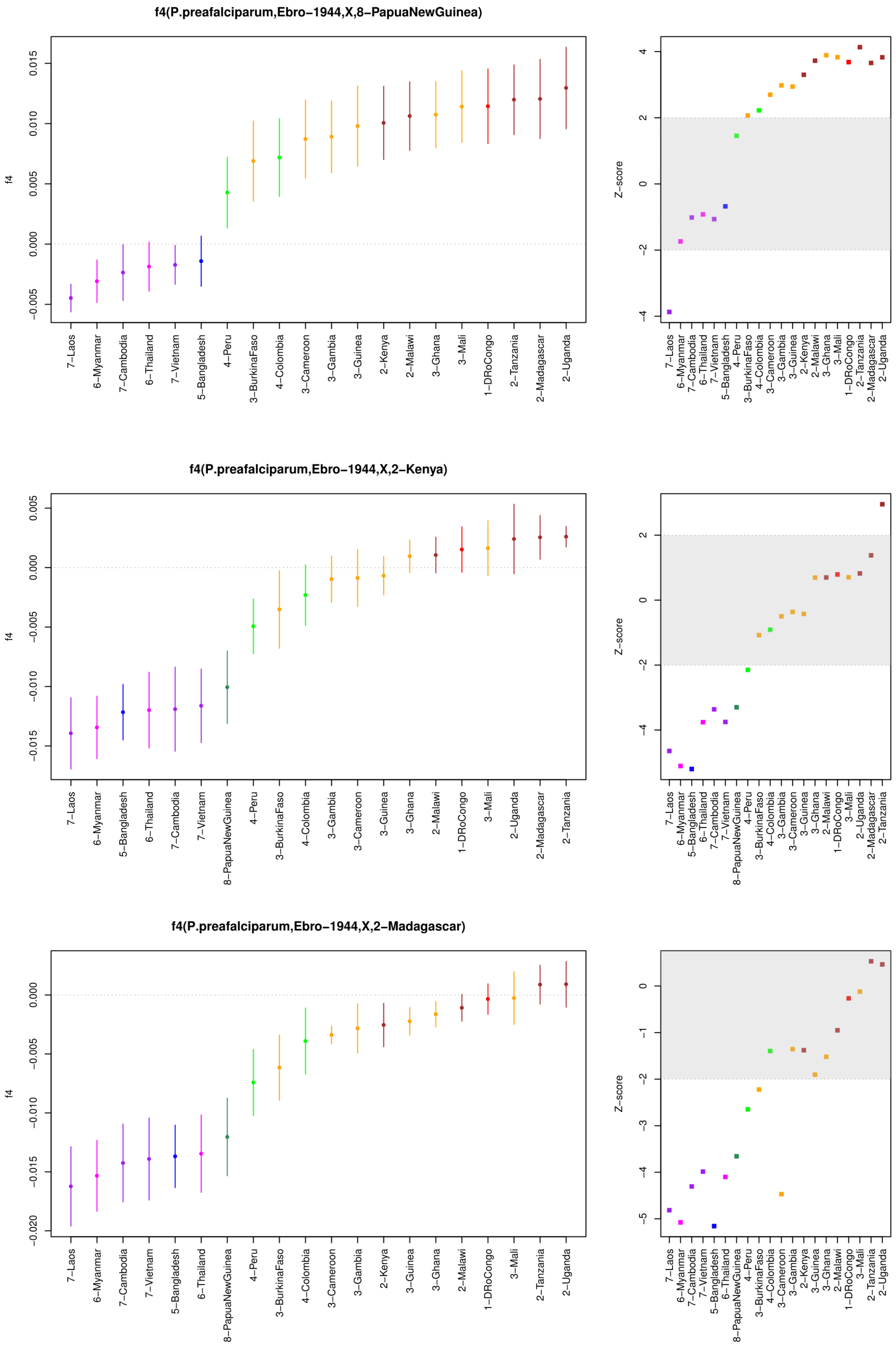
f4(P.preafalciparum,Ebro−1944,X,8−PapuaNewGuinea)
f4
Z−score
7−Laos 6−Myanmar 7−Cambodia 6−Thailand 7−Vietnam 5−Bangladesh 4−Peru 3−BurkinaFaso 4−Colombia 3−Cameroon 3−Gambia 3−Guinea 2−Kenya 2−Malawi 3−Ghana 3−Mali 1−DRoCongo 2−Tanzania 2−Madagascar 2−Uganda
f4(P.preafalciparum,Ebro−1944,X,2−Kenya)
f4
Z−score
7−Laos 6−Myanmar 5−Bangladesh 6−Thailand 7−Cambodia 7−Vietnam 8−PapuaNewGuinea 4−Peru 3−BurkinaFaso 4−Colombia 3−Gambia 3−Cameroon 3−Guinea 3−Ghana 2−Malawi 1−DRoCongo 3−Mali 2−Uganda 2−Madagascar 2−Tanzania
f4(P.preafalciparum,Ebro−1944,X,2−Madagascar)
f4
Z−score
7−Laos 6−Myanmar 7−Cambodia 7−Vietnam 5−Bangladesh 6−Thailand 8−PapuaNewGuinea 4−Peru 3−BurkinaFaso 4−Colombia 3−Cameroon 3−Gambia 2−Kenya 3−Guinea 3−Ghana 2−Malawi 1−DRoCongo 3−Mali 2−Tanzania 2−Uganda

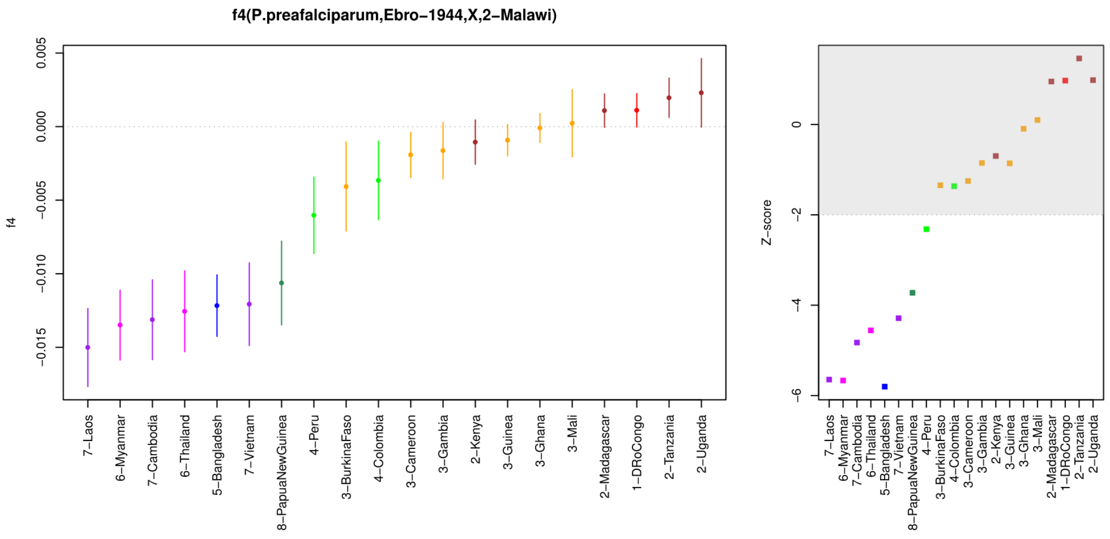
f4(P.preafalciparum,Ebro−1944,X,2−Malawi)
f4
Z−score
7−Laos
6−Myanmar
7−Cambodia
6−Thailand
5−Bangladesh
7−Vietnam
8−PapuaNewGuinea
4−Peru
3−BurkinaFaso
4−Colombia
3−Cameroon
3−Gambia
2−Kenya
3−Guinea
3−Ghana
3−Mali
2−Madagascar
1−DRoCongo
2−Tanzania
2−Uganda

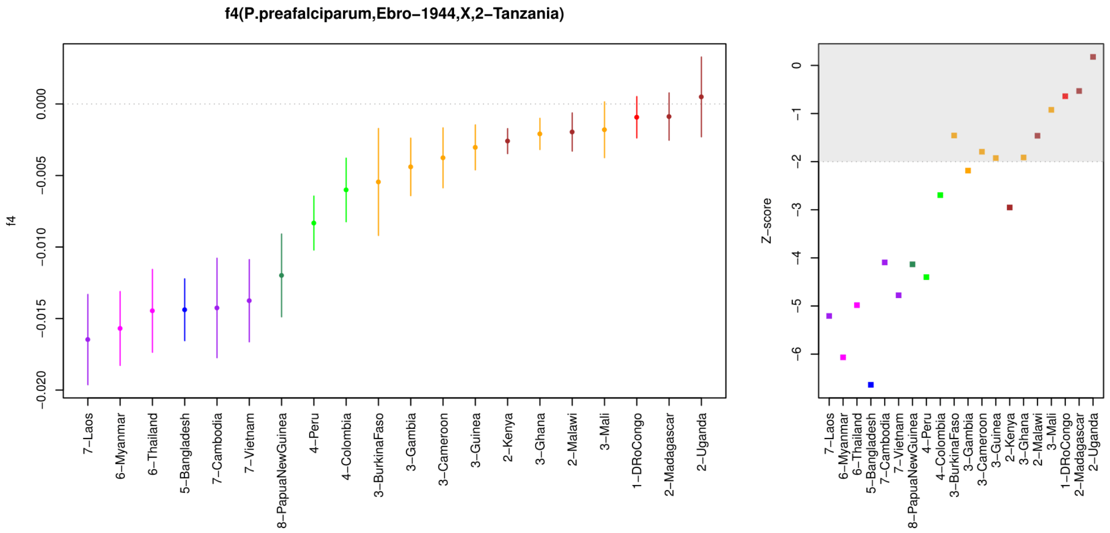
f4(P.preafalciparum,Ebro−1944,X,2−Tanzania)
f4
Z−score
7−Laos
6−Myanmar
6−Thailand
5−Bangladesh
7−Cambodia
7−Vietnam
8−PapuaNewGuinea
4−Peru
4−Colombia
3−BurkinaFaso
3−Gambia
3−Cameroon
3−Guinea
2−Kenya
3−Ghana
2−Malawi
3−Mali
1−DRoCongo
2−Madagascar
2−Uganda

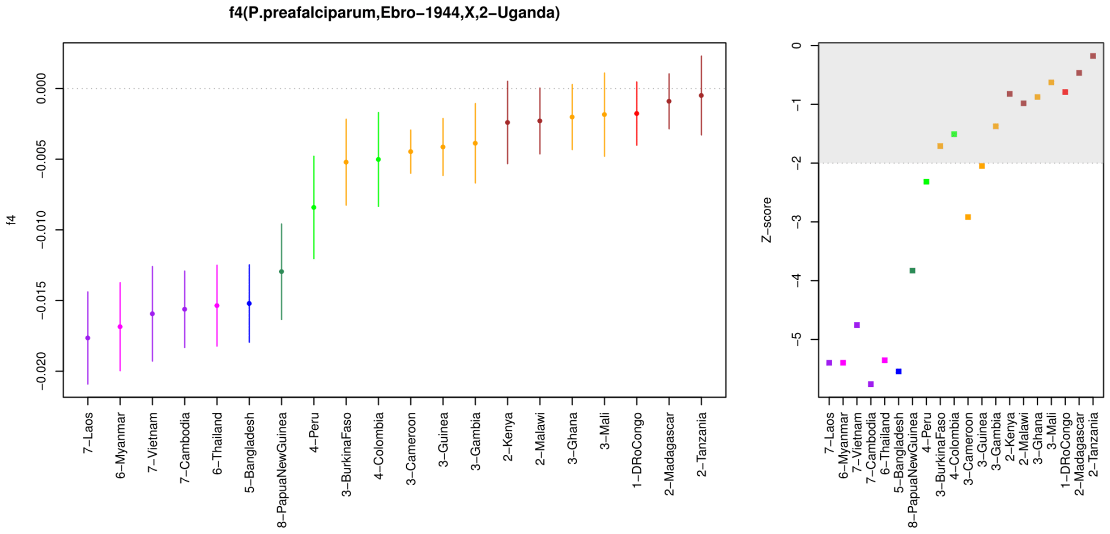
f4(P.preafalciparum,Ebro−1944,X,2−Uganda)
f4
Z−score
7−Laos
6−Myanmar
7−Vietnam
7−Cambodia
6−Thailand
5−Bangladesh
8−PapuaNewGuinea
4−Peru
3−BurkinaFaso
4−Colombia
3−Cameroon
3−Guinea
3−Gambia
2−Kenya
2−Malawi
3−Ghana
3−Mali
1−DRoCongo
2−Madagascar
2−Tanzania

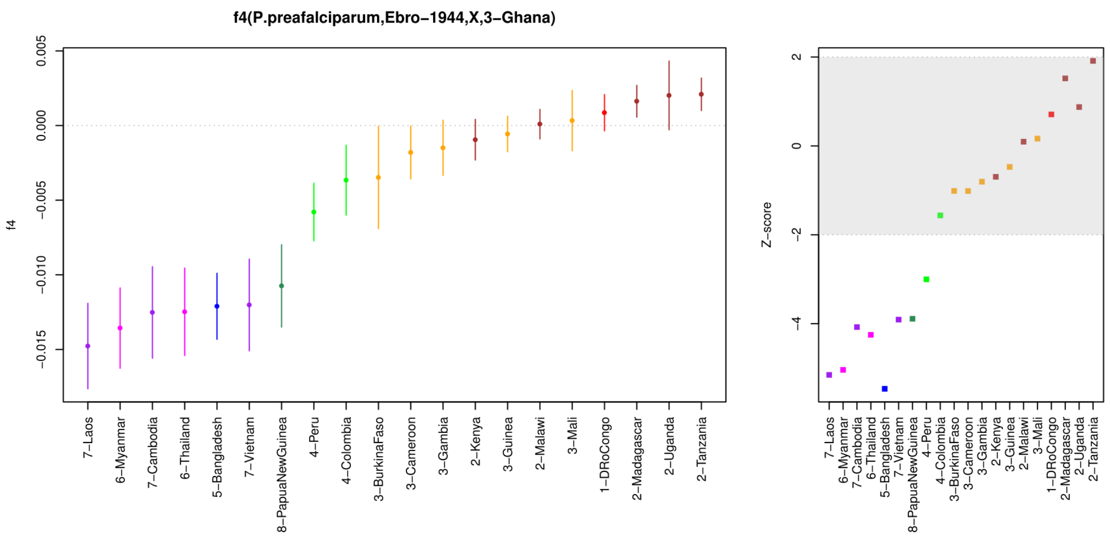
f4(P.preafalciparum,Ebro−1944,X,3−Ghana)
f4
Z−score
7−Laos
6−Myanmar
7−Cambodia
6−Thailand
5−Bangladesh
7−Vietnam
8−PapuaNewGuinea
4−Peru
4−Colombia
3−BurkinaFaso
3−Cameroon
3−Gambia
2−Kenya
3−Guinea
2−Malawi
3−Mali
1−DRoCongo
2−Madagascar
2−Uganda
2−Tanzania

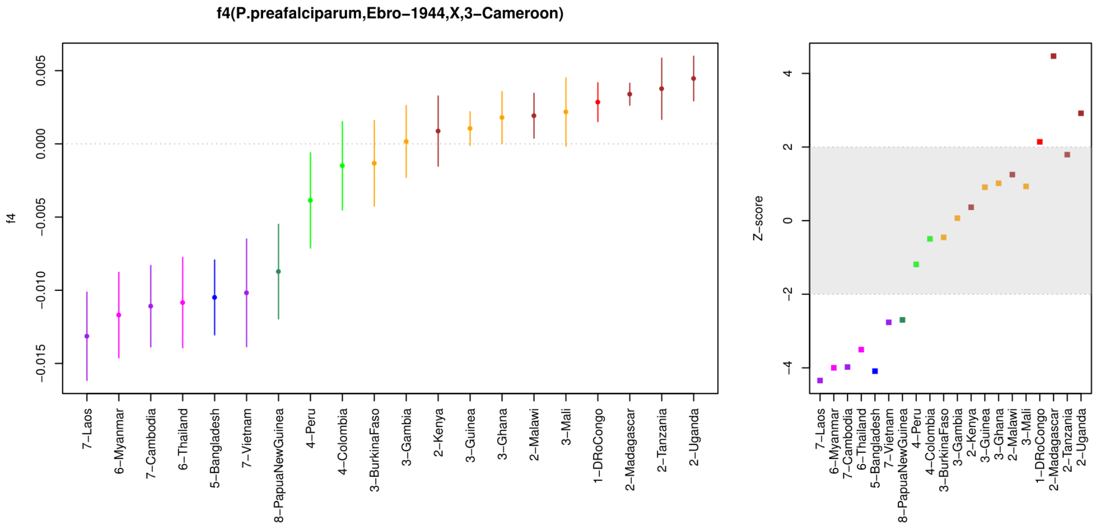
f4(P.preafalciparum,Ebro−1944,X,3−Cameroon)
f4
Z−score
7−Laos
6−Myanmar
7−Cambodia
6−Thailand
5−Bangladesh
7−Vietnam
8−PapuaNewGuinea
4−Peru
4−Colombia
3−BurkinaFaso
3−Gambia
2−Kenya
3−Guinea
3−Ghana
2−Malawi
3−Mali
1−DRoCongo
2−Madagascar
2−Tanzania
2−Uganda

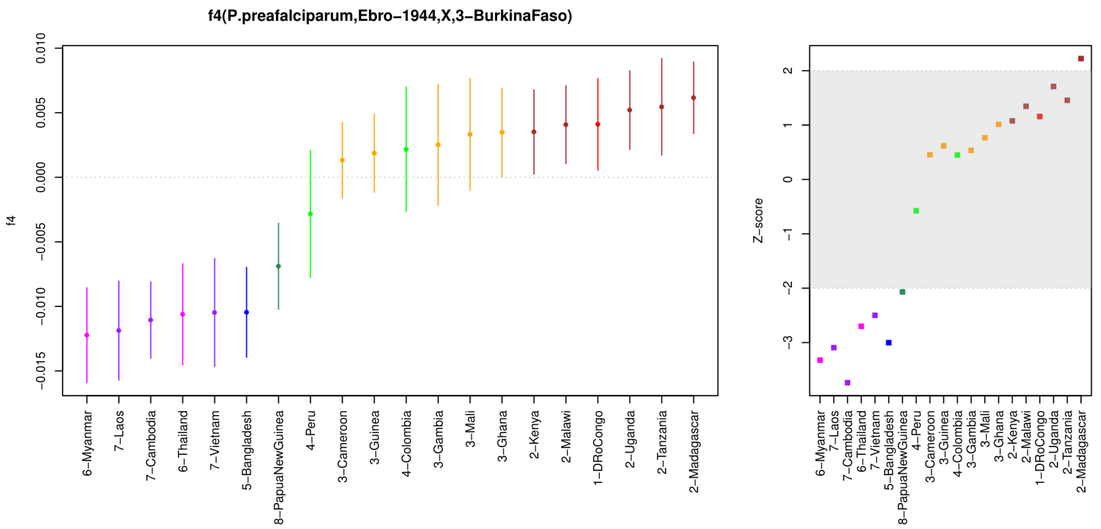
f4(P.preafalciparum,Ebro−1944,X,3−BurkinaFaso)
f4
Z−score
6−Myanmar
7−Laos
7−Cambodia
6−Thailand
7−Vietnam
5−Bangladesh
8−PapuaNewGuinea
4−Peru
3−Cameroon
3−Guinea
4−Colombia
3−Gambia
3−Mali
3−Ghana
2−Kenya
2−Malawi
1−DRoCongo
2−Uganda
2−Tanzania
2−Madagascar

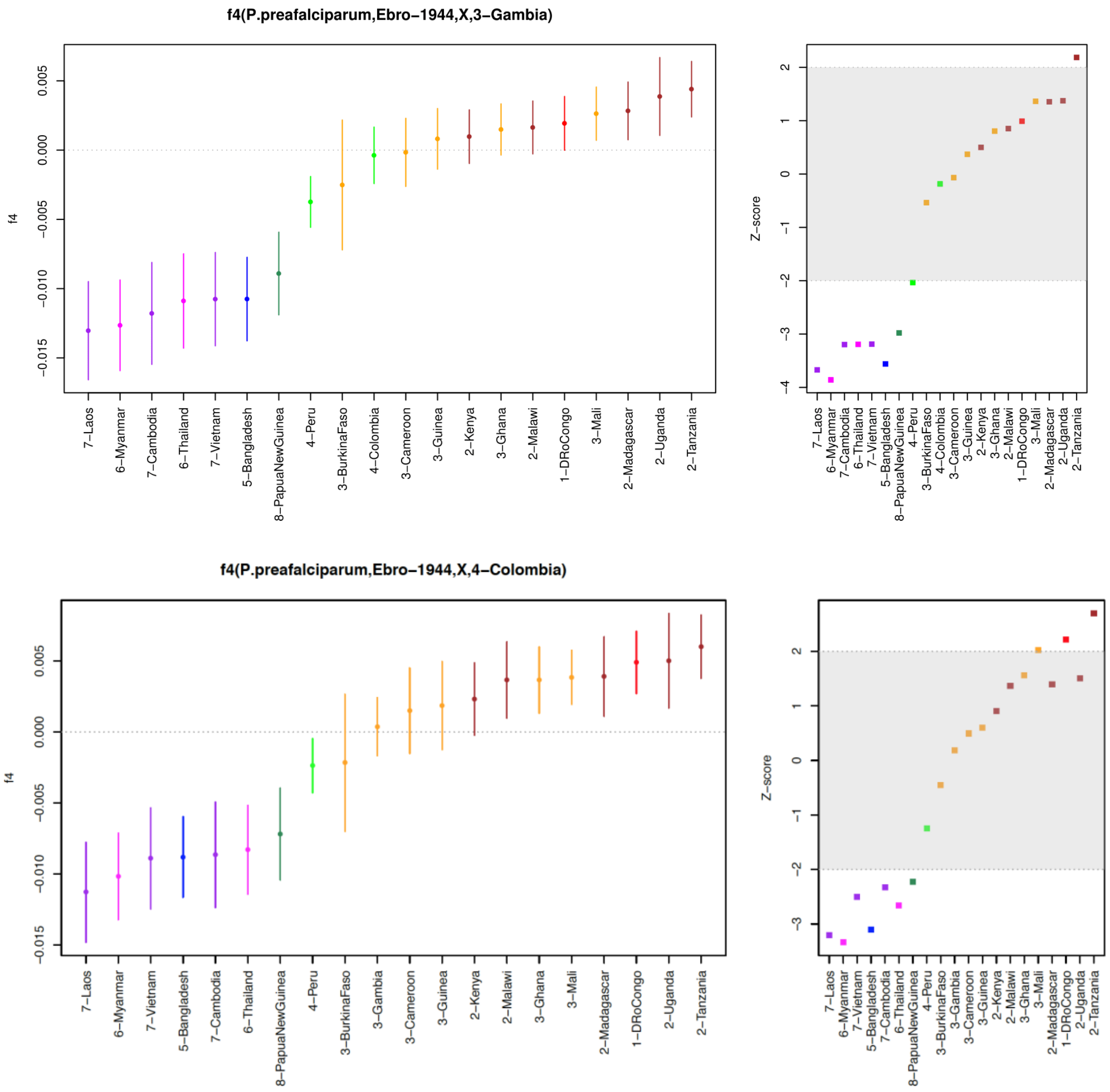

Figure S4: *f4*-statistics. (left-panel) *f4* statistics and 5-95% confidence intervals (y-axis) testing the relationship *f4*(*P. preafalciparum*, Ebro-1944; X, Y), where X and Y iterate through all groups included in our global dataset, as given in the header of each plot. (right-panel) Z-scores following jackknife resampling where an absolute Z-score greater than 2 is considered significant. Regional groupings are coloured as in Fig. 1 of the main text. A negative *f4* statistic indicates that Ebro-1944 has a greater affinity to X (x-axis label) over Y.

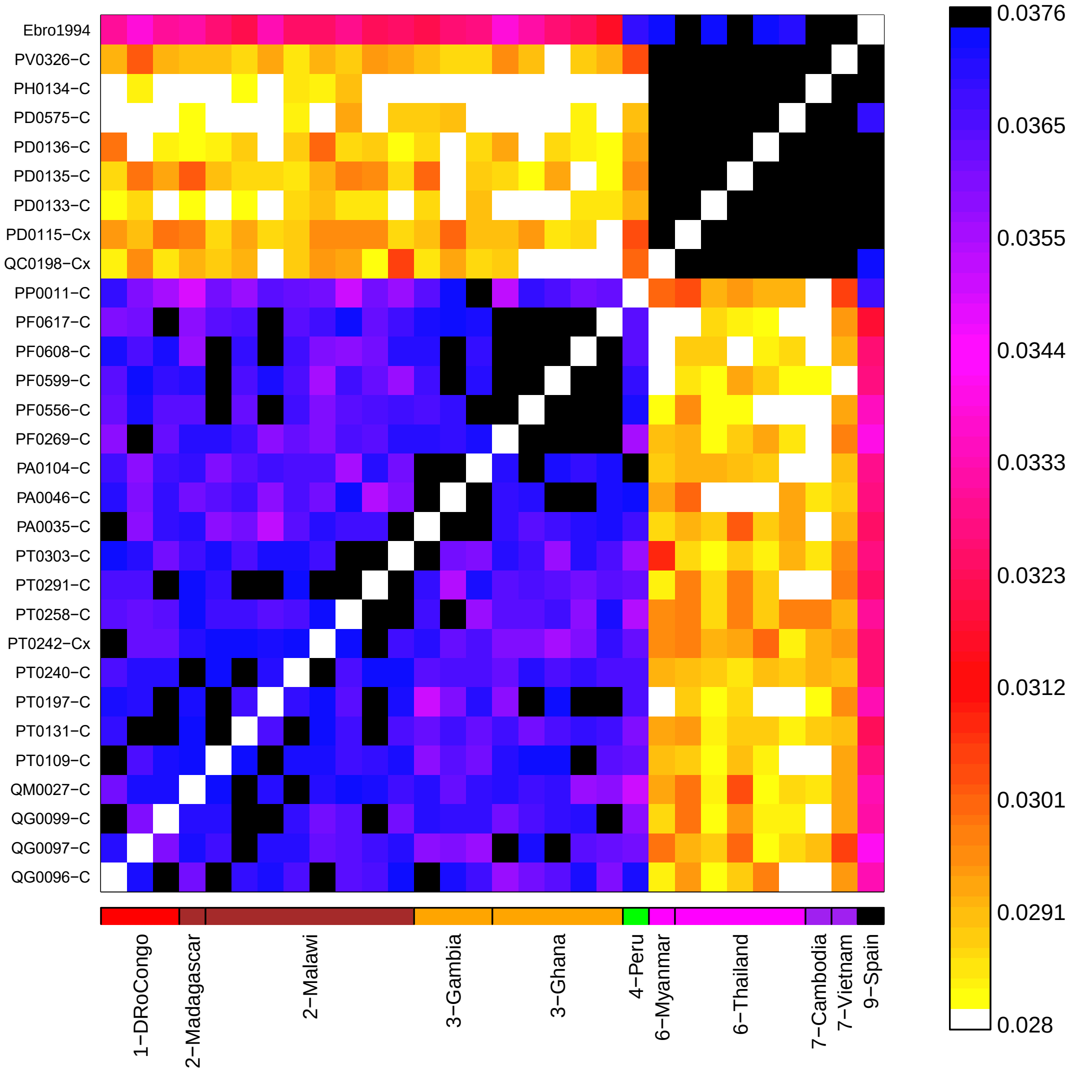

Figure S5: Chromopainter's inferred proportion of haplotypes shared (colour scale) between 30 strains of *P. falciparum* with 100% SNP overlap across 8195 variant sites. The x-axis colour provides the continental region where strains were collected. Ebro-1944 shares more haplotypes with strains from central south Asia relative to Africa.

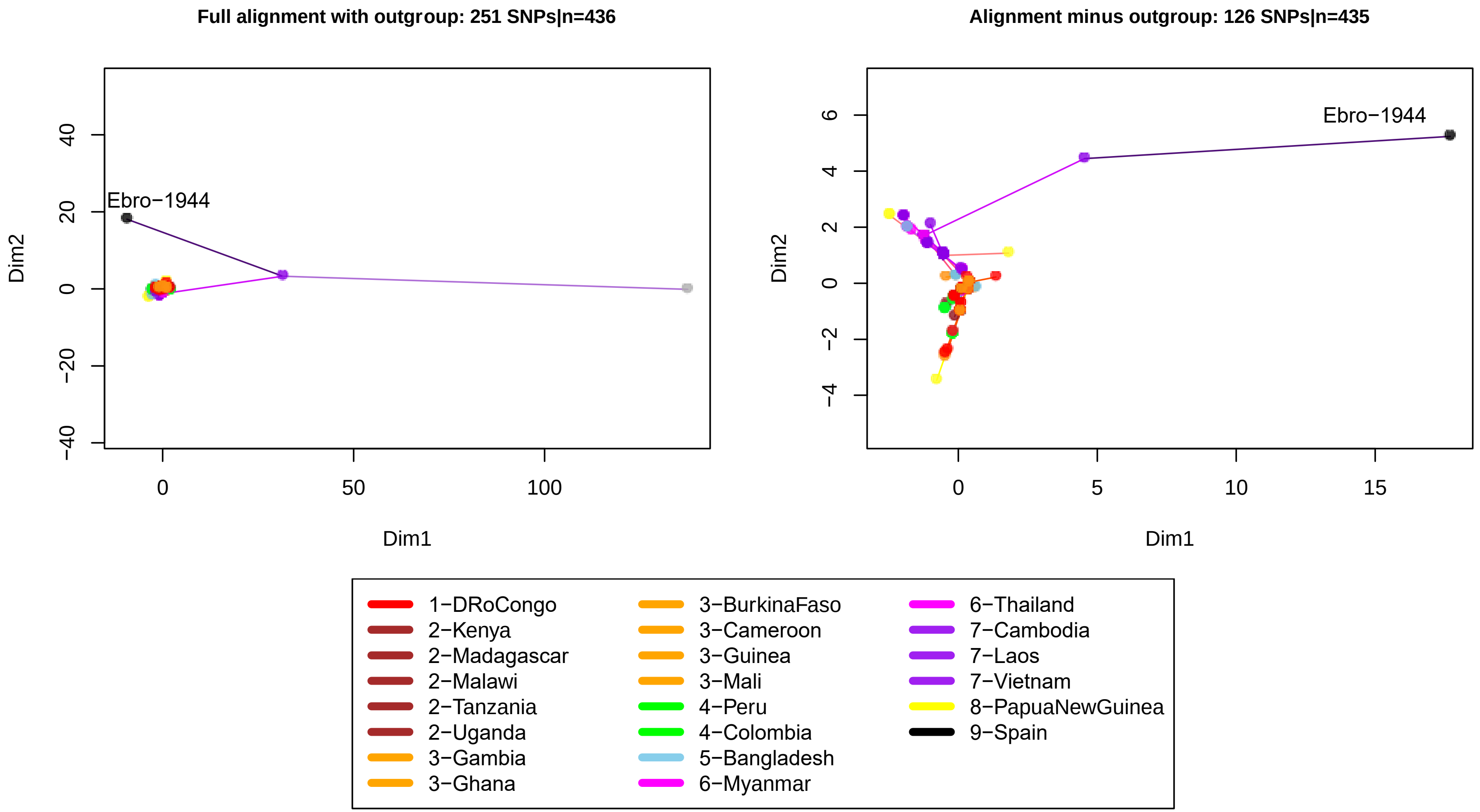

Figure S6: Minimum spanning network of the Ebro1944 mitochondrial genome.

Table S1: *Plasmodium falciparum* strains used in the population genetics analyses. See supplementary excel file.

Table S2: *Plasmodium falciparum* drug resistance variants described in the literature and screened in the Ebro-1944 strain. See supplementary excel file.

Table S3: mtDNA mutations overlapping in different slides. Distribution of the three geographically diagnostic mtDNA mutations across the four analysed slides. None of the ancestral variants are present in any slide, which suggests that the four slides contain a very similar *P. falciparum* strain. The nt276 and nt2763 positions are only found in Indian strains; the nt725 position is found in Indian and East African strains.

**mtDNA nt position**	**Slide CA**	**Slide CM**	**Slide POS**	**Slide Lane8**
276	Derived (N=12)	Derived (N=3)	Derived (N=8)	Derived (N=4)
725	Derived (N=8)	not covered	Derived (N=1)	Derived (N=3)
2763	Derived (N=23)	Derived (N=2)	not covered	Derived (N=5)

.

Supplementary References

1. Dabney J, Knapp M, Glocke I, Gansauge M-T, Weihmann A, Nickel B, et al. Complete mitochondrial genome sequence of a Middle Pleistocene cave bear reconstructed from ultrashort DNA fragments. Proc Natl Acad Sci U S A. 2013;110(39):15758–63.
2. March S, Ng S, Velmurugan S, Galstian A, Shan J, Logan DJ, et al. A microscale human liver platform that supports the hepatic stages of *Plasmodium falciparum* and *vivax*. Cell Host Microbe. 2013;14(1):104–15.
3. Meyer M, Kircher M. Illumina sequencing library preparation for highly multiplexed target capture and sequencing. Cold Spring Harb Protoc. 2010;5(6):pdb.prot5448.
4. Amato R, Miotto O, Woodrow CJ, Almagro-Garcia J, Sinha I, Campino S, et al. Genomic epidemiology of artemisinin resistant malaria. Elife. 2016;5:e08714.
5. Otto TD, Gilabert A, Crellen T, Böhme U, Arnathau C, Sanders M, et al. Genomes of all known members of a *Plasmodium* subgenus reveal paths to virulent human malaria. Nat Microbiol. 2018;3(6):687–97.
6. Helmby H, Cavelier L, Pettersson U, Wahlgren M. Rosetting Plasmodium falciparum-infected erythrocytes express unique strain-specific antigens on their surface. Infect Immun. 1993;61(1):284–8.
7. Baruch DI, Pasloske BL, Singh HB, Bi X, Ma XC, Feldman M, et al. Cloning the *P. falciparum* gene encoding PfEMP1, a malarial variant antigen and adherence receptor on the surface of parasitized human erythrocytes. Cell. 1995;82(1):77–87.
8. Oberli A, Slater LM, Cutts E, Brand F, Mundwiler-Pachlatko E, Rusch S, et al. A *Plasmodium falciparum* PHIST protein binds the virulence factor PfEMP1 and comigrates to knobs on the host cell surface. FASEB J. 2014;28(10):4420–33.

9. Nunes MC, Okada M, Scheidig-Benatar C, Cooke BM, Scherf A. *Plasmodium falciparum* fikk kinase members target distinct components of the erythrocyte membrane. PLoS One. 2010;5(7):e11747.

10. Goel S, Palmkvist M, Moll K, Joannin N, Lara P, Akhouri RR, et al. RIFINs are adhesins implicated in severe *Plasmodium falciparum* malaria. Nat Med. 2015;21:314.

11. McRobert L, Preiser P, Sharp S, Jarra W, Kaviratne M, Taylor MC, et al. Distinct trafficking and localization of STEVOR proteins in three stages of the *Plasmodium falciparum* life cycle. Infect Immun. 2004;72(11):6597–602.

12. Lavazec C, Sanyal S, Templeton TJ. Hypervariability within the Rifin, Stevor and Pfmc-2TM superfamilies in P*lasmodium falciparum*. Nucleic Acids Res. 2006; 34: 6696–6707.

13. Su X zhuan, Heatwole VM, Wertheimer SP, Guinet F, Herrfeldt JA, Peterson DS, et al. The large diverse gene family var encodes proteins involved in cytoadherence and antigenic variation of *Plasmodium falciparum*-infected erythrocytes. Cell. 1995;82(1):89–100.

14. Cheng Q, Cloonan N, Fischer K, Thompson J, Waine G, Lanzer M, et al. stevor and rif are *Plasmodium falciparum* multicopy gene families which potentially encode variant antigens. Mol Biochem Parasitol. 1998;97(1–2):161–76.

15. Fernandez V. Small, Clonally Variant Antigens Expressed on the Surface of the *Plasmodium falciparum*-infected Erythrocyte Are Encoded by the rif Gene Family and Are the Target of Human Immune Responses. J Exp Med. 2002;190(10):1393–404.

16. Kyes SA, Rowe JA, Kriek N, Newbold CI. Rifins: A second family of clonally variant proteins expressed on the surface of red cells infected with *Plasmodium falciparum*. Proc Natl Acad Sci U S A. 2002;96(16):9333–8.

17. Kaviratne M, Khan SM, Jarra W, Preiser PR. Small Variant STEVOR Antigen Is Uniquely Located within Maurer’s Clefts in *Plasmodium falciparum* -Infected Red

Blood Cells. Eukaryot Cell. 2002;1(6):926–35.

18. Niang M, Bei AK, Madnani KG, Pelly S, Dankwa S, Kanjee U, et al. STEVOR is a *Plasmodium falciparum* erythrocyte binding protein that mediates merozoite invasion and rosetting. Cell Host Microbe. 2014;16(1):81–93.

19. Spielmann T, Fergusen DJP, Beck H-P. etramps, a new *Plasmodium falciparum* gene family coding for developmentally regulated and highly charged membrane proteins located at the parasite-host cell interface. Mol Biol Cell. 2003;14(4):1529–44.

20. Winter G, Kawai S, Haeggström M, Kaneko O, von Euler A, Kawazu S, et al. SURFIN is a polymorphic antigen expressed on *Plasmodium falciparum* merozoites and infected erythrocytes. J Exp Med. 2005;201(11):1853–63.

21. Oksanen J, Blanchet FG, Friendly M, Roeland Kindt P, Legendre DM, Minchin PR, et al. Vegan: Community Ecology Package. R Package Version 2.2-0. 2019. Available at: http://CRAN.Rproject.org/package=vegan.

22. Sallares R, Gomzi S. Biomolecular archaeology of malaria. Ancient Biomolecules 2001; 3: 195–213.

23. Altschul SF, Gish W, Miller W, Myers EW, Lipman DJ. Basic local alignment search tool. J Mol Biol. 1990;215(3):403–10.

24. McKenna A, Hanna M, Banks E, Sivachenko A, Cibulskis K, Kernytsky A, et al. The Genome Analysis Toolkit: A MapReduce framework for analyzing next-generation DNA sequencing data. Genome Res. 2010;20(9):254–60.

25. Browning BL, Browning SR. Genotype Imputation with Millions of Reference Samples. Am J Hum Genet. 2016;98(1):116–26.

26. Broad Institute. *Plasmodium vivax* Hybrid Selection initiative [Internet].
